# A Dy^3+^ Single-Molecule Magnet as a Dual-Mode Luminescent and Magnetic Thermometer

**DOI:** 10.1021/acs.inorgchem.6c02533

**Published:** 2026-09-11

**Authors:** Paula Oreiro-Martínez, Cristina González-Barreira, Julio Corredoira-Vázquez, Ana M. García-Deibe, Jesús Sanmartín-Matalobos, Carlos D. S. Brites, Luis D. Carlos, Daniel Aravena, Matilde Fondo

**Affiliations:** ‡ Departamento de Química Inorgánica, Facultade de Química, 16780 Universidade de Santiago de Compostela, 15782 Santiago de Compostela, Spain; ε Institute of Materials (iMATUS), 16780 Universidade de Santiago de Compostela, 15782 Santiago de Compostela, Spain; § Phantom-g, CICECO−Aveiro Institute of Materials, Physics Department 56062 University of Aveiro, 3810-193, Aveiro, Portugal; ψ Departamento de Química de los Materiales, Facultad de Química y Biología, 428356 Universidad de Santiago de Chile, Casilla 40, Correo 33, Santiago, Chile

## Abstract

Metal-ion-assisted Schiff-base condensation enabled the isolation of the complex [Dy­(L^N6prop^)­(CH_3_COO)_2_]­(BPh_4_) (**1**), which undergoes a ligand exchange reaction with triphenylsilanolate to yield [Dy­(L^N6prop^)­(CH_3_COO)­(OSiPh_3_)]­(BPh_4_)·0.5CH_2_Cl_2_ (**2**·0.5CH_2_Cl_2_). The crystal structure of **2**·0.5CH_2_Cl_2_ reveals that the Dy^3+^ ion is coordinated in an *N*_6_ *O*_3_ environment, adopting a distorted capped square antiprism geometry. **2**·0.5CH_2_Cl_2_ is a single ion magnet, exhibiting magnetic relaxation in the 2–22 K range with an energy barrier of 465 K, consistent with *ab initio* calculations and luminescent measurements. **2**·0.5CH_2_Cl_2_ also functions as a luminescent thermometer with maximum sensitivity *S*_*r*_^Δ^ of 1% K^–1^ at 12 K. In addition, **2**·0.5CH_2_Cl_2_ also shows magneto-thermometry, based on magnetic relaxation time (τ) and inverse magnetic susceptibility (1/χ). This magneto-thermometer achieves relatively high magneto-thermal sensitivities (*S*_*max*_) of 9.3 % K^–1^ (*S*_*r*_^1/χ^) at 10 K and 20 % K^–1^ (*S*_*r*_^τ^) at 9 K, while both *S*_*r*_ parameters remain well above 1% K^–1^ across the entire single-molecule magnet (SMM) operating range. Overall, **2**·0.5CH_2_Cl_2_ constitutes a bifunctional molecular material, integrating SMM behavior with efficient dual-mode luminescent and magnetic thermometry.

## Graphical abstract

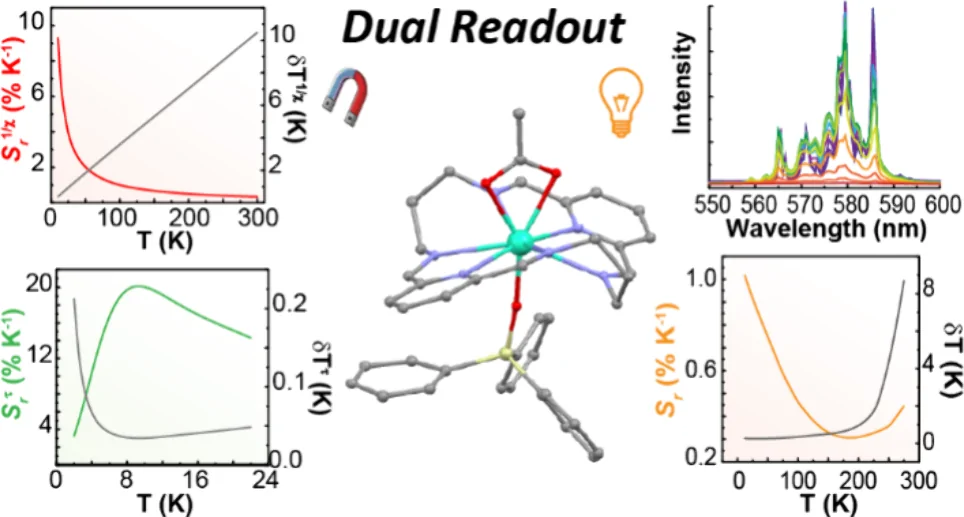

## Introduction

Single-molecule magnets (SMMs) constitute a paradigmatic class of bottom-up nanomaterials in which bistable magnetic states and slow magnetization dynamics arise intrinsically from the individual molecules. Since their discovery,^1^ these systems have received considerable attention owing to their potential in areas such as high-density data storage, spintronic and quantum technologies.^2–4^ Although remarkable progress has been achieved in enhancing magnetic anisotropy barriers and blocking temperatures, particularly in lanthanoid-based systems,^5–7^ several critical challenges must still be addressed before SMMs can be integrated into functional nanoscale devices.

Among these challenges, the control and accurate determination of temperature at the nanometric scale have emerged as central issues. The magnetic dynamics of SMMs are extremely sensitive to temperature, as thermally activated processes, quantum tunneling of magnetization, and spin–phonon coupling jointly can govern magnetic relaxation.^8,9^ At the nanoscale, conventional macroscopic thermometry becomes inadequate, highlighting the need for local, molecule-compatible temperature probes.

In this context, luminescent thermometry offers a powerful, non-invasive approach to nanoscale temperature sensing.^10^ Besides, strong magnetic anisotropy and characteristic *4f*–*4f* luminescence can coexist within the same lanthanoid center. This intrinsic coupling between optical and magnetic properties can enable real-time monitoring of local temperature under conditions relevant to SMM operation.^7,11,12^

Nevertheless, the synthesis of SMMs that simultaneously exhibit outstanding magnetic properties and pronounced thermal sensitivity in their luminescence continues to be challenging. This difficulty arises from the dissimilar electronic requirements underlying both properties. In lanthanoid-based systems, magnetic blocking and luminescent thermometric performance are both governed, among other parameters, by the crystal-field (CF) splitting of the Ln^3+^ electronic levels, which is closely related to the symmetry of the coordination environment. For instance, in Dy^3+^ SMMs, luminescent thermometry typically relies on the thermal population of crystal-field sublevels derived from the emitting ^4^F_9/2_ state. A smaller energy separation between these CF sublevels leads to higher thermal responsiveness of luminescence at low temperatures.^13^ However, reduced coordination symmetry, which favors smaller CF splitting, also decreases the separation between the ground and first excited CF sublevels, thereby lowering the effective magnetic anisotropy barrier *U*_eff_ and compromising magnetic performance.^9,14,15^ Consequently, an intrinsic conflict exists between the structural requirements needed to optimize both magnetic blocking and luminescent thermometry.

Several approaches have been reported in the literature to address this limitation, including the design of SMMs in which the magnetic properties originate from the lanthanoid ion, while the thermometric luminescence is provided by the ligand framework.^7^ This strategy has enabled the realization of the only SMM reported to date that operates as a luminescence-based thermometer below the SMM blocking temperature. Nevertheless, the resulting thermometric performance remains limited below 40 K, as the relative thermal sensitivity achieved is rather low.

Thus, alternative approaches based on magnetic thermometry are also emerging.^16,17^ In this sense, magnetic observables such as static susceptibility or relaxation dynamics are directly correlated with temperature, enabling nanoscale thermometric readout without relying on optical emission. Notably, Dy^3+^-based SMMs exhibiting thermal sensitivities as high as 11.9 % K^–1^ have recently been reported using this approach,^17^ while sensitivities of up to *ca*. 70 % K^–1^ have been achieved by employing a multiple linear regression (MLR) strategy that integrates independent magnetic and luminescent thermometric responses.^17^

With these considerations in mind, and building on our previous experience in SMMs featuring luminescent thermometry,^7,18–20^ herein we report the magnetic performance and luminescent-, magneto- and multiparametric-thermometric properties of a dysprosium single-ion magnet based on an *N*_6_ macrocyclic ligand, highlighting its multifunctional behavior and potential for nanoscale temperature sensing.

## Experimental Section

### General

All chemical reagents were commercially sourced and employed as received, without any additional purification.

Elemental analyses of C, H and N were carried out using a Thermoscientific Flash Smart analyser. FT-IR spectra were collected with a Varian FT-IR 670 spectrometer fitted with an attenuated total reflectance (ATR) accessory over the 500–4000 cm^–1^ spectral range.

### Syntheses

#### {[Dy­(L^N6prop^)­(CH_3_COO)_2_]­(BPh_4_)} (1)

A mixture of 2,6-pyridinedicarboxaldehyde (0.150 g, 1.110 mmol) and dysprosium­(III) acetate tetrahydrate (0.228 g, 0.555 mmol) is magnetically stirred in dry methanol (40 mL) for 15 min. Then, 1,3-propylenediamine (99%, 0.087 g, 1.110 mmol) is added, and the resulting mixture is refluxed for 24 h. A pale-yellow solution is obtained, to which sodium tetraphenylborate (99%, 0.192 g, 0.555 mmol) is added, precipitating a white solid. The suspension is stirred for 2 h and then concentrated to dryness using a rotary evaporator. The resulting product is washed with 50 mL of water, filtered, and dried in an oven. Yield: 0.411 g (78%). MW: 946.243 g/mol. Elemental analysis (%) experimental: C 60.79, N 8.80, H 5.19; calculated for C_48_H_48_BDyN_6_O_4_: C 60.93, N 8.88, H 5.11. IR (ATR, ν̃/cm^–1^): 1652 (νCN_imine_), 1590 (νCN_Py_), 1540, 1444 (νCOO_acetate_).

#### {[Dy­(L^N6prop^)­(CH_3_COO)­(OSiPh_3_)]­(BPh_4_)}·0.5CH_2_Cl_2_ (2·0.5CH_2_Cl_2_)

Triphenylsilanol (98%, 0.086 g, 0.305 mmol) and sodium hydride (60%, 0.012 g, 0.305 mmol) are dissolved in dry tetrahydrofuran (5 mL) with magnetic stirring under an argon atmosphere. The resulting triphenylsilanolate solution is then added to compound **1** (0.150 g, 0.153 mmol), previously dissolved in 15 mL of CH_2_Cl_2_ under an argon atmosphere, and stirred for 24 h. The resulting suspension is centrifuged, and the solid is separated and washed with water (50 mL). The white solid obtained is dried in an oven and recrystallized from dichloromethane by diethyl ether diffusion. This leads to single crystals of **2**·0.5CH_2_Cl_2_, suitable for X-ray diffraction studies. Yield: 0.075 g (42%). MW: 1205.0 g/mol. Elemental analysis (%) experimental: C 64.37, N 6.98, H 5.10; calculated for DyC_64.5_H_61_BClN_6_O_3_Si: C 64.29, N 6.97, H 5.10. IR (ATR, ν̃/cm^–1^): 1660 (νCN_imine_), 1590 (νCN_Py_), 1540, 1450 (νCOO_acetate_), 993 (νSiO).

### Crystal Structure Analyses

Since the crystals of **2**·0.5CH_2_Cl_2_ were complexly twinned, and despite being carefully selected, a piece of one of the twins had to be measured. Diffraction data were acquired at 100(2) K, using monochromatized Mo Kα radiation, λ = 0.71073 Å, on a Bruker D8 Venture Photon III-14 diffractometer. The resulting data were processed, and absorption effects were corrected using a multi-scan procedure with the TWINABS routine.^21^ The presence of twinned reflections prevented the completion of a full data set, but the solution of the molecular structure was entirely satisfactory. The crystal structure was solved using conventional direct methods with SHELXT,^22^ and subsequently refined with SHELXL,^23^ by full-matrix least-squares procedures based on *F* ^2^. Anisotropic displacement parameters were assigned to all non-hydrogen atoms during refinement. Most hydrogen atoms were positioned geometrically and incorporated into the structure-factor calculations using a riding model, with their displacement parameters defined relative to those of the corresponding parent atoms. Further crystallographic and refinement details are provided in Table S1.

CCDC 2542650 contains the supplementary crystallographic data for this paper. These data can be obtained free of charge from the Cambridge Crystallographic Data Centre.

### Powder X-ray Diffraction Studies

Powder X-ray diffraction measurements for **2**·0.5CH_2_Cl_2_ were recorded on a Philips-type diffractometer equipped with a “PW1710” control unit, a vertical goniometer “PW1820/00”, and an “Enraf Nonius FR590” generator operating at 40 kV and 30 mA. X-rays were produced using a sealed Cu tube, and the radiation was monochromatized with a graphite monochromator (λCuKα = 1.5406 Å). Powder X-ray diffraction patterns were recorded over a 2θ interval of 2–30°, using a step increment of 0.02° and an acquisition time of 3 s per step. During data collection, the sample was continuously rotated to improve the quality of the diffraction profiles and reduce possible preferred-orientation effects. To minimize background scattering associated with conventional glass substrates, the sample was deposited on an oriented Si(511) single-crystal plate. To ensure that the powder diffraction measurements were not affected by preferred orientation, the sample was ground in a mortar prior to mounting on the diffractometer. The resulting diffraction pattern was analyzed using HighScore Plus software (Version 3.0d).

### Magnetic Measurements

*dc* and *ac* magnetic measurements for **2**·0.5CH_2_Cl_2_ were collected using a Quantum Design SQUID MPMS3 susceptometer. The temperature-dependent *dc* susceptibility was measured from 2 to 300 K under an applied field of 0.1 T, while magnetization measurements under fields ranging from 0 to 7 T were collected at a fixed temperature of 2 K. Diamagnetic contributions were corrected using Pascal’s Tables. Alternating current (*ac*) experiments were conducted under both zero *dc* field and an applied *dc* field of 1000 Oe, using an oscillating field amplitude of 3.5 Oe over the frequency range of 1–1000 Hz. Magnetic hysteresis loops were measured between −3 and +3 T at temperatures from 2 to 10 K, employing a field sweep rate of 100 Oe/s.

### Luminescence Measurements

Solid-state emission and excitation spectra of **2**·0.5CH_2_Cl_2_ were recorded as a function of temperature using a Fluorolog3 spectrofluorometer (FL3-2T, Horiba). The system was equipped with a modular double grating excitation monochromator (fitted with a 1200 grooves per mm grating blazed at 330 nm) and a TRIAX 320 single emission monochromator (fitted with a 1200 grooves per mm grating blazed at 500 nm, reciprocal linear density of 2.6 nm mm^–1^), coupled with a photomultiplier (R298, Hamamatsu), using a front-face measurement configuration. Excitation was provided by a 450 W Xe arc lamp. The recorded emission profiles were adjusted to compensate for the detector response and the spectrofluorometer optical characteristics, while the excitation spectra were corrected for the spectral distribution of the lamp intensity with a photodiode reference detector. The temperature was controlled using a helium closed-cycle cryostat, a vacuum system (4×10^–4^ Pa), and an autotuning temperature controller (Lakeshore 330, Lakeshore) with a resistance heater. The temperature was measured with a silicon diode cryogenic sensor (DT-470-SD, Lakeshore), providing accuracies of ±0.5 K (12–30 K), ±0.25 K (30–60 K), and ±0.15 K (60–340 K).

### Electronic Structure Calculations

CASSCF^24^ calculations were performed using the ORCA 6 software package.^25^ The basis set was SARC2-DKH-QZVP for Dy^26^ and the relativistic decontracted version of Def2-TZVP.^27^ Scalar and spin-orbit relativistic effects were described by the DKH2 Hamiltonian^28,29^ and the Quasi-degenerate perturbation theory (QDPT), respectively. Tunneling relaxation times based on the dipolar model^30^ were calculated using the *UandTau* software.^31^ f-orbital energies are obtained on top of CASSCF results by means of the *ab initio* ligand field theory (AILFT) approach.^32^

## Results and Discussion

### Synthesis and Structural Characterization

[Dy­(L^N6prop^)­(CH_3_COO)_2_]­(BPh_4_) (**1**) was obtained by a modification of a published method,^33,34^ according to Scheme 1. Its reaction with triphenylsilanol and sodium hydride in 1:1 molar ratio and subsequent recrystallization in CH_2_Cl_2_ lead to the isolation of [Dy­(L^N6prop^)­(CH_3_COO)­(OSiPh_3_)]­(BPh_4_)·0.5CH_2_Cl_2_ (**2**·0.5CH_2_Cl_2_) as single crystals.

**Scheme 1:**
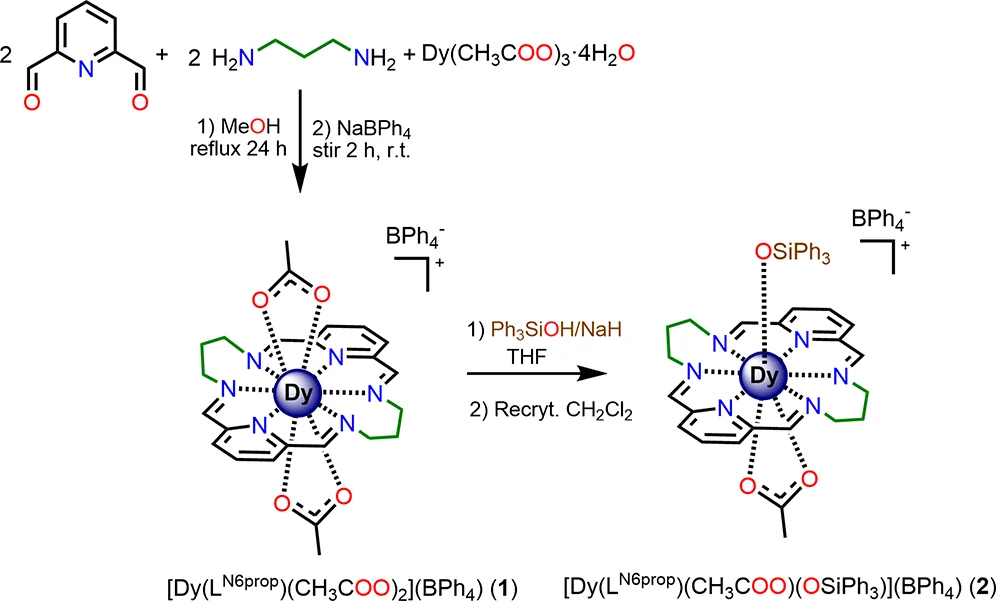
Isolation of Precursor **1** and Complex **2** ^a^

a Solvate molecules are omitted for clarity.

The two complexes were characterized using elemental analysis and infrared (IR) spectroscopy. Additionally, **2**·0.5CH_2_Cl_2_ was characterized by single-crystal and powder X-ray techniques, as well as by magnetic measurements. Analysis of this complex as a moleculer magnet and as a luminescent, magnetic, and multiparametric thermometer was also performed.

### X-ray Diffraction Studies

Selected experimental data obtained from the single-crystal X-ray diffraction analyses for **2**·0.5CH_2_Cl_2_ are summarized in Table S1.

**2**·0.5CH_2_Cl_2_ is an ionic compound, consisting of [Dy­(L^N6prop^)­(CH_3_COO)­(OSiPh_3_)]^+^ cations, [BPh_4_]^−^ anions, and CH_2_Cl_2_ solvates. Figure 1 shows an ellipsoid diagram of the cation, and Table S2 records the main bond lengths and angles.

**Figure 1:**
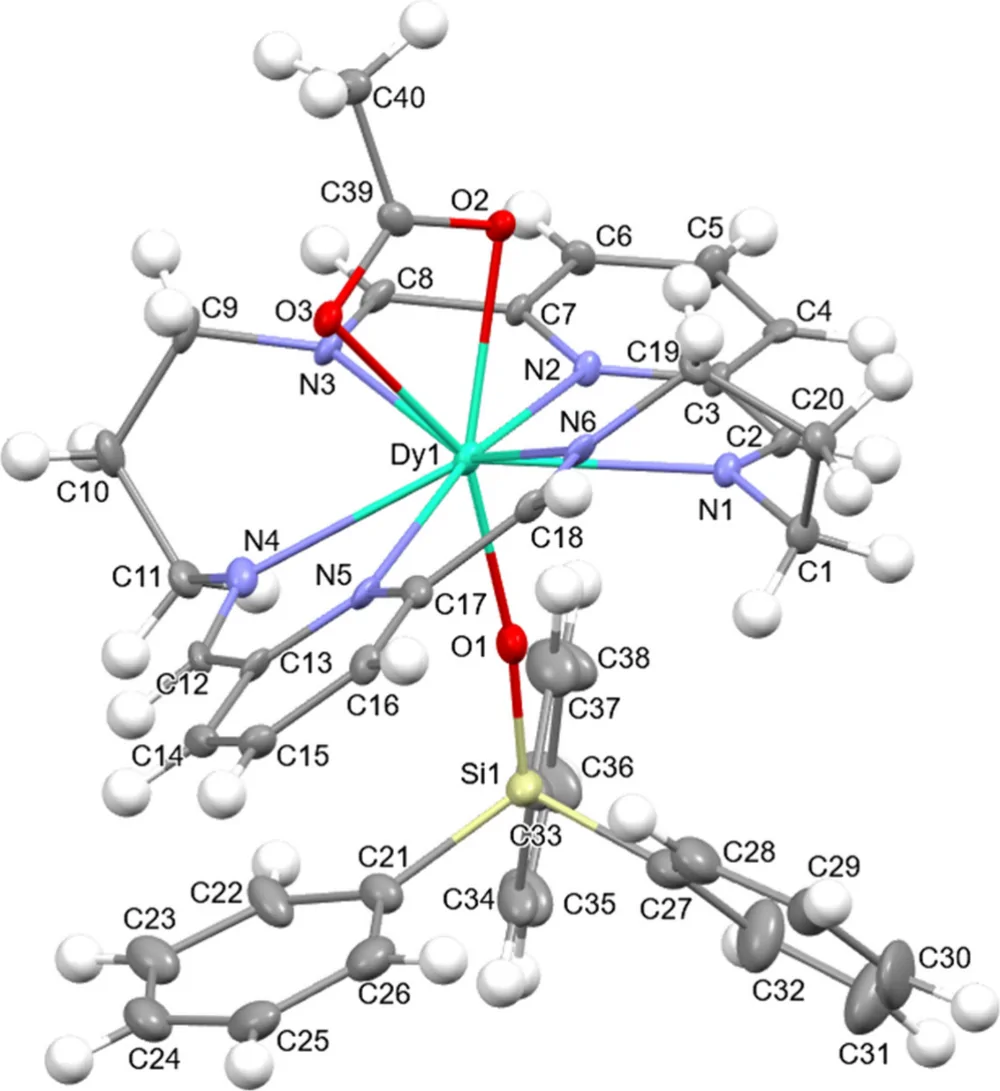
Ellipsoids diagram for the cation {[Dy­(L^N6prop^)­(CH_3_COO)­(OSiPh_3_)]}^+^ in **2**·0.5CH_2_Cl_2_.

The structural characterization reveals that the macrocyclic ligand acts as a hexadentate *N*_6_ donor in the [Dy­(L^N6prop^)­(CH_3_COO)­(OSiPh_3_)]^+^ cation, coordinating to the dysprosium ion through its six nitrogen atoms (two pyridine and four imine). These nitrogen atoms define a distorted plane around the Dy^3+^ ion, as illustrated in Figure 1. The deviations of the N atoms from the least-squares *N*_6_ plane range from 0.140 to 0.766 Å, with the smallest deviations observed for the pyridine nitrogen donors, while the dysprosium atom is displaced by 0.11 Å from this plane.

Additionally, the coordination sphere of the lanthanoid center is further completed by a bidentate chelate acetate ligand and a terminal monodentate triphenylsilanolate donor. As a result, the Dy^3+^ center is in an *N*_6_ *O*_3_ environment, corresponding to a coordination number of 9. Calculations performed with SHAPE^35^ agree with a distorted capped square antiprismatic tending toward a muffin shape geometry (Table S3) for the metal ion.

The crystal packing of adjacent molecules indicates that the Dy^3+^ ions are well isolated, with the shortest Dy^3+^···Dy^3+^ distance being *ca*. 13.3 Å.

Furthermore, powder X-ray diffraction measurements were performed for **2**·0.5CH_2_Cl_2_ (Figure S1). The data show that the experimental pattern is in good agreement with the expected crystalline phase and does not display any additional reflections, confirming the high phase purity of the isolated material.

### Electronic Structure Calculations

Prior to the analysis of the magnetic properties, CASSCF calculations were performed using the crystallographic coordinates for **2**. These indicate that the ground state is markedly anisotropic, showing a large easy axis *g*-factor (*g*_*z*_ = 19.857) together with small transverse components (*g*_*x*_ = 0.005 and *g*_*y*_ = 0.006). This is consistent with a relatively slow ground-state quantum tunneling of magnetization (4.2 × 10^–4^ s). The first excited state lies 325 cm^–1^ above the ground state and is expected to limit Orbach relaxation since this Kramers’ doublet already presents sizable transverse components of *g* (Table 1).

**Table 1:** CASSCF Calculated Energies and Spin-Hamiltonian Parameters for the Stark Sublevels of the ^6^H_15/2_ Multiplet

KD	*E* (cm^–1^)	*g*_*x*_	*g*_*y*_	*g*_*z*_
1	0.0	0.005	0.006	19.857
2	324.6	0.283	0.455	16.756
3	496.5	2.848	4.726	11.562
4	591.4	3.560	5.161	8.786
5	719.4	0.127	2.289	15.300
6	756.0	1.132	1.922	12.742
7	783.8	0.102	1.081	17.800
8	903.2	0.060	0.162	18.411

The energies of the f-orbitals were also calculated, and they show that the highly repulsive triphenylsilanol ligands define the splitting pattern, as the two most destabilized orbitals are a mixture of f_0_ (f_*z* ^3^_) and one of the f_±1_ orbitals, which point toward the position of the silanol coordination with energies equal to 1126 cm^–1^ and 1183 cm^–1^ (Figure 2a). The remaining f_±1_ orbitals lie at an intermediate energy of 667 cm^–1^. Orbitals oriented in the *xy* plane (the f_±2_ and f_±3_ blocks) appear at energies below 400 cm^–1^, favoring the oblate arrangement of the Dy^3+^ ion. In this way, the easy magnetic axis points parallel to the Dy–O_silanolate_ bond, with a deviation lower than 1° (Figure 2b).

**Figure 2:**
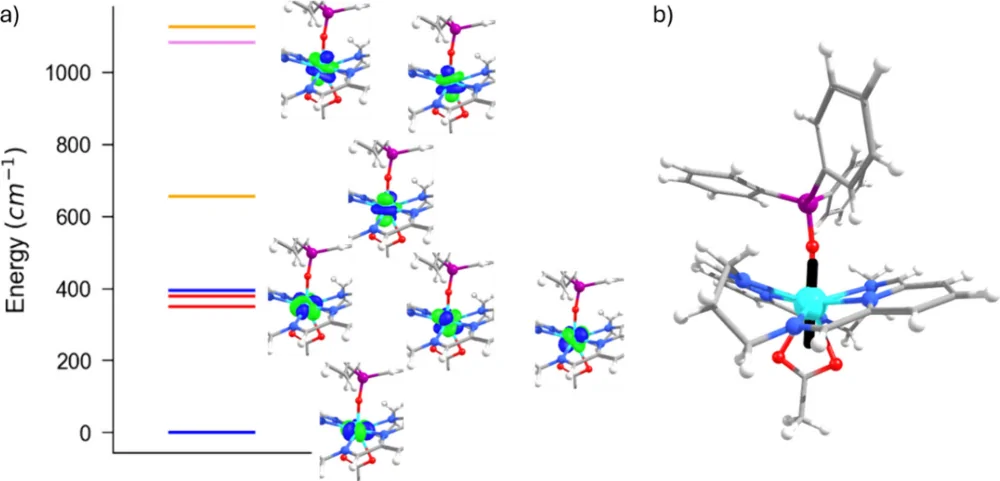
(a) AILFT-calculated f-orbital energies and (b) orientation of *g*_*z*_ for the ground state of 2 (black). Color code: Dy (light blue); Si (violet); O (red); N (blue); C (gray); H (white).

### Magnetic Properties

Magnetic susceptibility data were obtained from a sample of **2**·0.5CH_2_Cl_2_ in the range 2–300 K (Figure S2), and variable-field magnetization measurements were also performed at 2 K, between 0 and 7 T (Figure S2, inset). As shown in Figure S2, the χ_M_ *T* product reaches 14.2 cm^3^ K mol^–1^ at 300 K, a value in close agreement with that expected for an isolated Dy^3+^ ion (14.17 cm^3^ K mol^–1^). Upon cooling, χ_M_ *T* gradually decreases down to *ca*. 15 K, followed by a more pronounced decline down to 2 K, consistent with the presence of significant magnetic anisotropy.

Furthermore, a steep rise in magnetization is observed in the low-field region, followed by a slow, nearly linear rise, reaching 5.7 Nμ_B_ at 7 T. This value falls well below the theoretical saturation moment calculated for a Dy^3+^ ion (10 Nμ_B_), pointing to pronounced magnetic anisotropy and an Ising-type ground-state doublet.^36^

The dynamic magnetic properties of **2**·0.5CH_2_Cl_2_ were also studied. Thus, *ac* susceptibility measurements were recorded in the absence of an external magnetic field. The χ″_M_ data exhibit clear temperature- (Figure S3) and frequency-dependent maxima between 2 and 22 K (Figure 3), consistent with the identification of **2**·0.5CH_2_Cl_2_ as a single-ion magnet (SIM).

**Figure 3:**
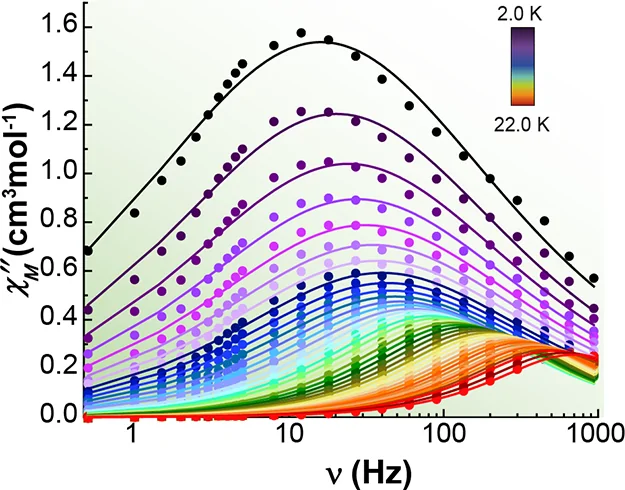
Frequency dependence of χ″_M_ (*H*_dc_ = 0) at different temperatures for **2**·0.5CH_2_Cl_2_.

Moreover, the Cole–Cole plot (Figure S4) displays curves with values of the distribution α parameter between 0.48 and 0.05. A high value of the distribution parameter α reflects a broader distribution of relaxation times (α = 0.48), which progressively narrows upon heating as a single dominant relaxation channel emerges (α = 0.05 at 22 K). This behavior is well documented in the literature for Dy^3+^-based SMMs and related lanthanoid systems.^37^

The temperature dependence of the relaxation time was used to characterize the relaxation dynamics (Figure 4). The experimental data were fitted by considering the possible relaxation pathways, excluding the direct mechanism, namely the Orbach, Raman, and QTM processes, either individually or in combination, according to eq 1.

$$\tau^{-1}={\tau_{0}}^{-1}\;e^{-U_{\mathrm{eff}}/k_{B}T}+{\mathrm{CT}}^{n}+{\tau_{\mathrm{QTM}}}^{-1}$$ 1

**Figure 4:**
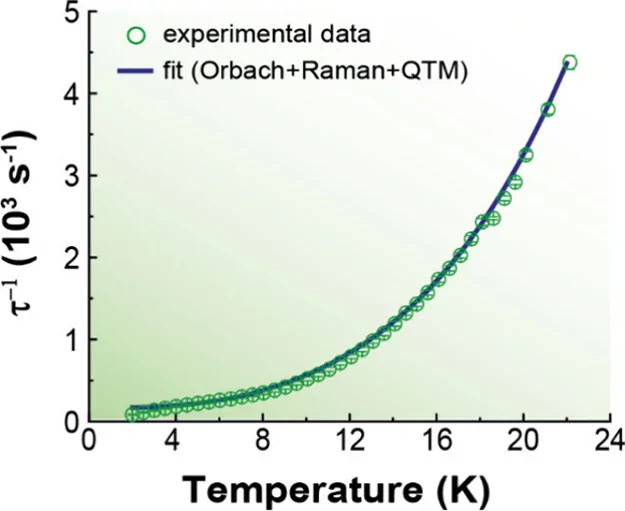
Dependence of the relaxation time with temperature for **2**·0.5CH_2_Cl_2_. The solid line represents the best fit to the experimental data (*r* ^2^ > 0.9999) considering Orbach plus Raman plus QTM relaxation processes.

In this fit, according to *ab initio* calculations, the energy barrier was restricted between 400 and 500 K. A satisfactory fit to the experimental data (Figure 4) is achieved taking into account all the relaxation pathways, and this renders the parameters *U*_eff_ = (465.3 ± 10.5) K, τ_0_ = (1.920 ± 0.005) × 10^–12^ s, *C* = (0.624 ± 0.001), *n* = (2.832 ± 0.006), and τ_QTM_ = (6.646 ± 0.05) × 10^–3^ s.

The *U*_eff_ value (323 cm^–1^) closely matches the barrier estimated from the *ab initio* calculations (325 cm^–1^). Besides, the presence of QTM is consistent with the shape of the χ″_M_ vs *T* graph, where the curves do not approach zero (Figure S3). Moreover, the calculated QTM contribution is in reasonable agreement with the fitted value. The remaining difference between the calculated and experimental tunneling times is likely due to the approximations inherent in the theoretical model, which neglect effects such as hyperfine and/or intermolecular interactions.

However, the obtained Raman exponent (*n* ≈ 2.83) may appear relatively low. Nevertheless, deviations from the ideal value predicted for Kramers’ ions (*n* = 9) can be rationalized by considering the combined contributions of acoustic and optical phonons to the magnetic relaxation,^38,39^ and *n* values as small as 2 were reported for other Dy^3+^ mononuclear complexes.^40^

Comparison of the energy barrier of **2**·0.5CH_2_Cl_2_ with those of other related [Dy­(L^N6R^)­(CH_3_COO)­(OSiPh_3_)]­X complexes (R = enMe or PhenMe, X = PF_6_ or BPh_4_) seems to reveal some emerging trends (Table S4).^41,42^ Thus, related [Dy­(L^N6enMe^)­(CH_3_COO)­(OSiPh_3_)]­(PF_6_)·3H_2_O,^41^ with electron-donating methyl substituents on the imine carbon atoms (Table S4), does not behave as a SIM in the absence of an applied magnetic field; however, under a *dc* field of 500 Oe, it displays an energy barrier of *ca*. 36 K, significantly lower than that observed for **2**·0.5CH_2_Cl_2_. In contrast, [Dy­(L^N6phenMe^)­(CH_3_COO)­(OSiPh_3_)]­(BPh_4_)·CH_3_OH, with methyl-substituted phenyl links between the imine-pyridine moieties (Table S4), exhibits SIM behavior at zero applied field,^42^ with a barrier (150 K) substantially lower than the one of **2**·0.5CH_2_Cl_2_.

A comparison of the relevant geometric parameters among these three complexes (Table S4) indicates that no single structural feature governs the magnitude of the energy barrier; rather, it arises from the combined influence of several parameters, as previously inferred for hexagonal bipyramidal (hbp) SMMs.^43^ In this context, [Dy­(L^N6enMe^)­(CH_3_COO)­(OSiPh_3_)]­(PF_6_)·3H_2_O shows the shortest Dy–N bond lengths, consistent with a stronger equatorial crystal field, but it also presents the shortest Dy–O distances, suggesting a comparatively strong axial field. Conversely, **2**·0.5CH_2_Cl_2_ shows the weakest equatorial and axial crystal fields, whereas [Dy­(L^N6phenMe^)­(CH_3_COO)­(OSiPh_3_)]­(BPh_4_)·CH_3_OH exhibits intermediate values of Dy–N and Dy–O distances. The significantly higher energy barrier for **2**·0.5CH_2_Cl_2_ seems to suggest that weakening the equatorial crystal field is a key factor, as the presence of a stronger axial field in related complexes (Table S4) does not lead to an improvement in the energy barrier. However, this correlation should be considered as a plausible structural trend rather than as a definitive design principle, since multiple structural and electronic parameters vary simultaneously when comparing the different Dy complexes. Thus, in agreement with previous observations for hbp complexes, the relaxation barrier is likely governed by the combined influence of several structural and electronic factors rather than from a single predominant parameter.^43^ Within this context, reducing the donor strength of the N atoms in the macrocyclic ligand appears to have a clearly beneficial effect on enhancing the energy barrier, not only in related octacoordinated complexes,^43,44^ but also in nonacoordinated *N*_6_ *O*_3_ macrocyclic compounds such as the one described herein.

Attempts to eliminate the observed QTM in **2**·0.5CH_2_Cl_2_ were made. Accordingly, additional measurements were performed under an optimized weak *dc* magnetic field of 1000 Oe (Figure S5). In this case, **2**·0.5CH_2_Cl_2_ shows a dependence of χ″_M_ on frequency (Figure 5 and S6), with maxima for the out of-phase susceptibility between 7 and 28 K.

**Figure 5:**
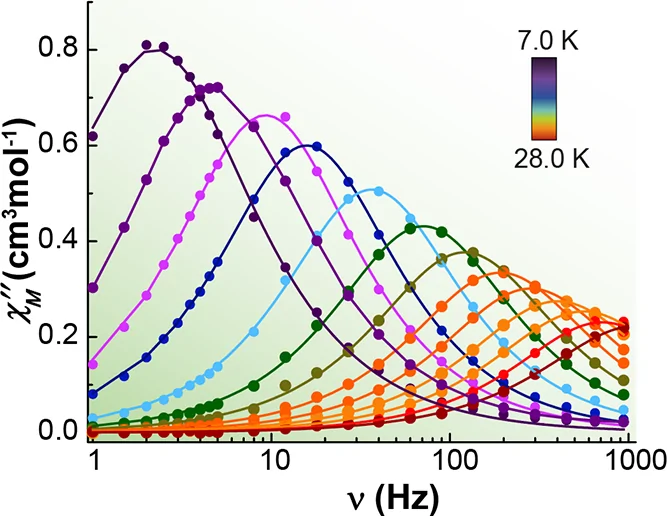
Frequency dependence of χ″_M_ at different temperatures for **2**·0.5CH_2_Cl_2_ in *H*_dc_ = 1000 Oe.

The new Cole–Cole plot (Figure S7) shows lower α values, ranging from 0.1 to 0.04, suggesting a narrow distribution of relaxation times. The τ^–1^ vs *T* graph under the 1000 Oe field (Figure 6) was then fitted considering once again all the terms of eq 1. The best fit is achieved with Orbach and Raman relaxation and renders the parameters *U*_eff_ = (466 ± 9.8) K, τ_0_ = (3.54 ± 0.80) × 10^–11^ s, *C* = (0.0987 ± 0.001), and *n* = (3.270 ± 0.009), showing that the applied field suppresses the zero-field tunneling channel within the experimental time scale.

**Figure 6:**
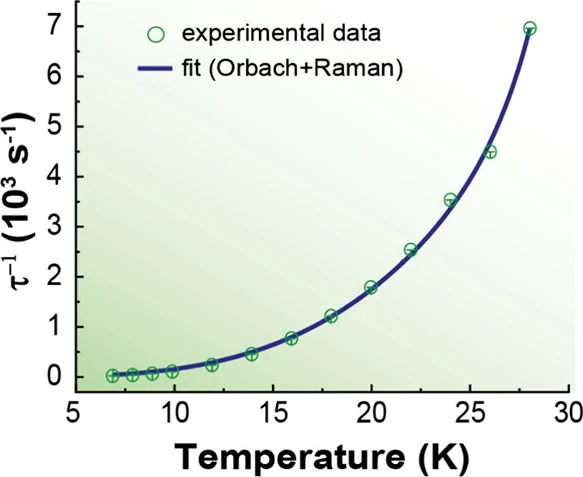
Temperature dependence of the relaxation time for **2**·0.5CH_2_Cl_2_ in *H*_dc_ = 1000 Oe. The solid line corresponds to the best fit (*r* ^2^ > 0.999) obtained using a combination of Orbach and Raman relaxation mechanisms.

Hysteresis loops for **2**·0.5CH_2_Cl_2_ were also recorded to test magnetic blocking. A characteristic butterfly-shaped hysteresis loop was observed under applied *dc* magnetic fields ranging from −3 to 3 T (Figure 7) at 2 K but not at higher temperatures. However, the absence of coercivity for **2**·0.5CH_2_Cl_2_ at zero field is consistent with the presence of an intrinsic fast QTM pathway at low temperatures, as it was inferred from *ac* measurements and *ab initio* calculations.

**Figure 7:**
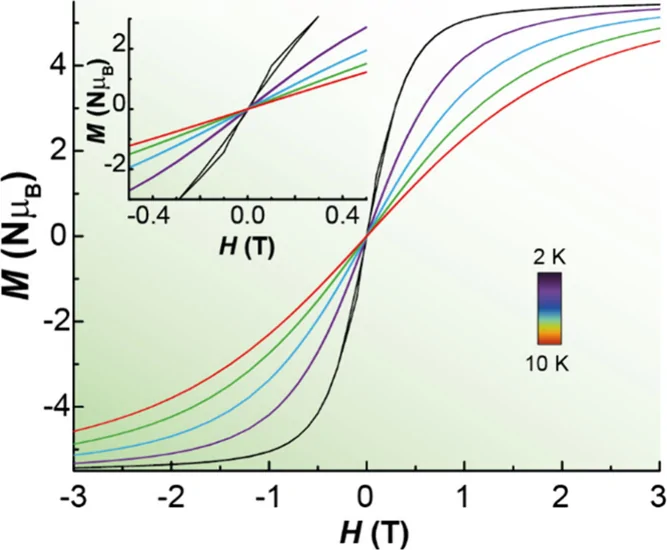
Hysteresis loops for **2**·0.5CH_2_Cl_2_, recorded in the 2–10 K temperature interval with a 100 Oe/s sweep rate. Inset: Expansion of the region near zero field.

### Luminescence Studies

The excitation spectra of **2**·0.5CH_2_Cl_2_ (12-300 K), recorded by monitoring the Dy^3+^ ^4^F_9/2_→^6^H_13/2_ transition, reveal a broad ligand-centered band in the 325–400 nm range (Figure S8). The emission spectra of **2**·0.5CH_2_Cl_2_ (Figure 8), recorded using an excitation wavelength of 348 nm, display the characteristic Dy^3+^ transitions from ^4^F_9/2_ to ^6^H_9/2_, ^6^H_11/2_, ^6^H_13/2_, and ^6^H_15/2_.

**Figure 8:**
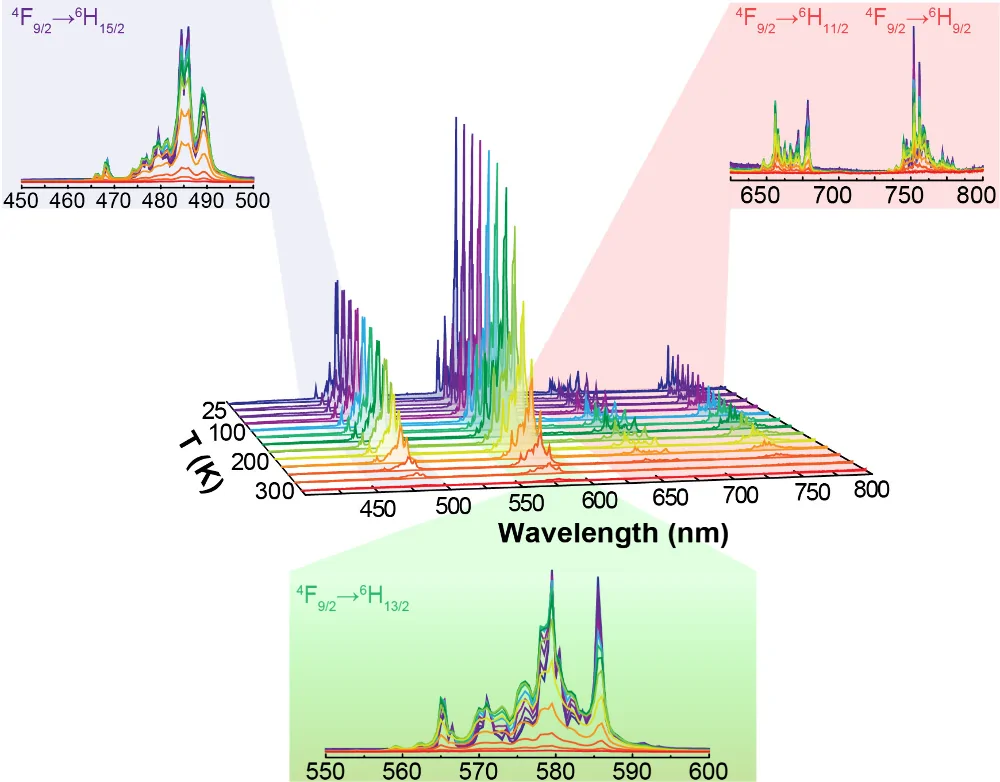
Emission spectra of **2**·0.5CH_2_Cl_2_ (12–300 K) upon 348 nm excitation, with a magnified view of the Dy^3+^ transitions showing the temperature dependence.

The ^4^F_9/2_→^6^H_15/2_ transition was deconvoluted to extract more detailed information (Figure S9 and Table S5). As Dy^3+^ is a Kramers ion, the maximum crystal field splitting of a level into Kramers doublets (KDs) is given by (2*J* + 1)/2. For the ^6^H_15/2_ multiplet, a maximum of eight KDs is therefore expected. The low symmetry of the Dy^3+^ local environment is expected to enhance the crystal-field splitting and facilitate the experimental resolution of the Stark components.

However, the presence of at least 12 spectral components indicates additional contributions beyond those arising from transitions involving only the lowest emitting KD. These additional features are most plausibly attributed to hot-band transitions originating from thermally populated excited KDs of the ^4^F_9/2_ emitting state.^16,45^ Furthermore, a contribution from resolved vibronic fine structure cannot be excluded.^46^

The deconvolution allows the determination of the relative energy of each KD. The results reveal an energy gap of 331.7 ± 0.6 cm^–1^ between the first two KDs of the ^6^H_15/2_ ground state. This value is in excellent agreement with that obtained from *ab initio* calculations (325 cm^–1^) and from experimental magnetic data (325 cm^–1^). Thus, the similarity between the luminescence-derived crystal-field splitting and the experimentally determined effective barrier further supports the interpretation that the thermally activated Orbach relaxation pathway proceeds through the first excited Kramers doublet, rather than involving higher-lying excited states.

### Thermometric Analyses

To evaluate the thermometric performance of **2**·0.5CH_2_Cl_2_, its temperature-dependent magnetic and luminescence responses were investigated.

Two intrinsic magnetic observables were employed as thermometric parameters: the inverse magnetic susceptibility (1/χ), derived from *dc* measurements, together with the magnetic relaxation time (τ), extracted from *ac* susceptibility data.

1/χ exhibits a linear dependence with temperature over the 10–300 K range (Figure 9a), following the Curie law (1/χ = *T*/*C*),^47^ with a Curie constant *C* = 14.11 ± 0.03 cm^3^ mol^–1^ K. This confirms the paramagnetic behavior of the system and the effective isolation of the magnetic ground state.^16^

**Figure 9:**
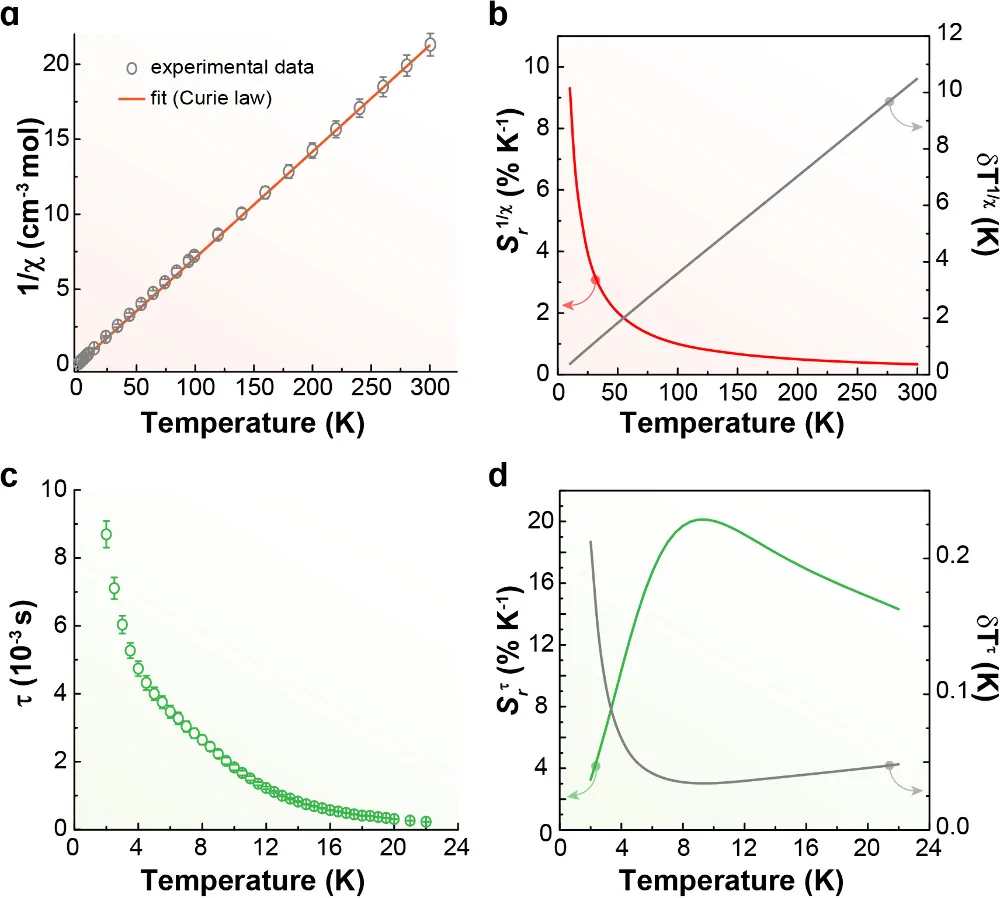
(a) Temperature variation of the reciprocal magnetic susceptibility (1/χ) measured over the 2–300 K temperature range with an applied field of 1000 Oe. The orange line denotes the Curie-law fit obtained for *T* > 10 K (*r* ^2^ > 0.999). (b) Temperature-dependent relative sensitivity (*S*_*r*_^1/χ^) and corresponding temperature uncertainty (δ*T* ^1/χ^) calculated from the inverse susceptibility data. (c) Temperature dependence (2–22 K) of the relaxation time (τ) measured under zero *dc* field. (d) Temperature dependence of the relative sensitivity (*S*_*r*_^τ^) and temperature uncertainty (δ*T* ^*τ*^) obtained from τ.

The performance of luminescent thermometers is commonly assessed using the relative sensitivity *S*_*r*_ and the temperature uncertainty, also referred to as temperature resolution (δ*T*).^48,49^ These parameters provide useful metrics for comparing thermometric performance across systems with different chemical environments.^50^ The thermal relative sensitivity (*S*_*r*_) can be expressed as follows:

$$S_{r}=\frac{1}{\Delta }\left|\frac{\partial \Delta }{\partial T}\right|$$ 2

On the other hand, δ*T* is expressed as

$$\delta T=\frac{1}{S_{r}}\frac{\delta \Delta }{\Delta }$$ 3

where δΔ/Δ denotes the relative error associated with the determination of the thermometric parameter.

The corresponding relative thermal sensitivity, *S*_*r*_^1/χ^, increases markedly upon decreasing temperature (Figure 9b), reaching values close to 9% K^–1^ at low temperatures (10 K), highlighting its suitability as a thermometric parameter in the cryogenic regime (*S*_*r*_^1/*χ*^ > 1% K^–1^ from 10 to 100 K). The temperature uncertainty associated with 1/χ, δ*T* ^1/χ^, was estimated from the experimental dispersion of the *dc* data. It exhibits a linear dependence on temperature, reaching a minimum value of *ca*. 0.3 K at 10 K, and below 1 K in the SMM operating regime (Figure 9b).

The τ parameter shows a strong temperature dependence within the low-temperature region (Figure 9c), decreasing progressively as temperature increases, consistent with thermally activated relaxation processes characteristic of SMM behavior. As a result, the relative thermal sensitivity derived from τ, *S*_*r*_^*τ*^, presents a pronounced maximum around 9 K, attaining values close to 20% K^–1^, significantly exceeding those obtained from 1/χ in this temperature range.^16,17^ This demonstrates that τ constitutes a highly sensitive thermometric parameter within the SMM operative regime (until 22 K), with *S*_*r*_^*τ*^ values higher than 3.5% K^–1^ throughout the entire SMM range. The temperature uncertainty associated with τ was estimated considering the standard deviations of the relaxation times obtained from a simple fit of the generalized Debye model, showing a temperature dependence and reaching its lowest value of *ca*. 0.03 K at 9 K. Remarkably, **2**·0.5CH_2_Cl_2_ displays the highest τ-based thermometric sensitivity reported to date (Table S6).

In parallel with the magnetic study, the luminescent properties were analyzed to evaluate their thermometric potential. All four transitions exhibit a similar temperature dependence (Figure 8). Therefore, the main ^4^F_9/2_→^6^H_13/2_ transition was chosen as representative of the luminescent thermometric performance of **2**·0.5CH_2_Cl_2_ because of its higher signal-to-noise ratio (Figure 10a), resulting in lower temperature uncertainty.

**Figure 10:**
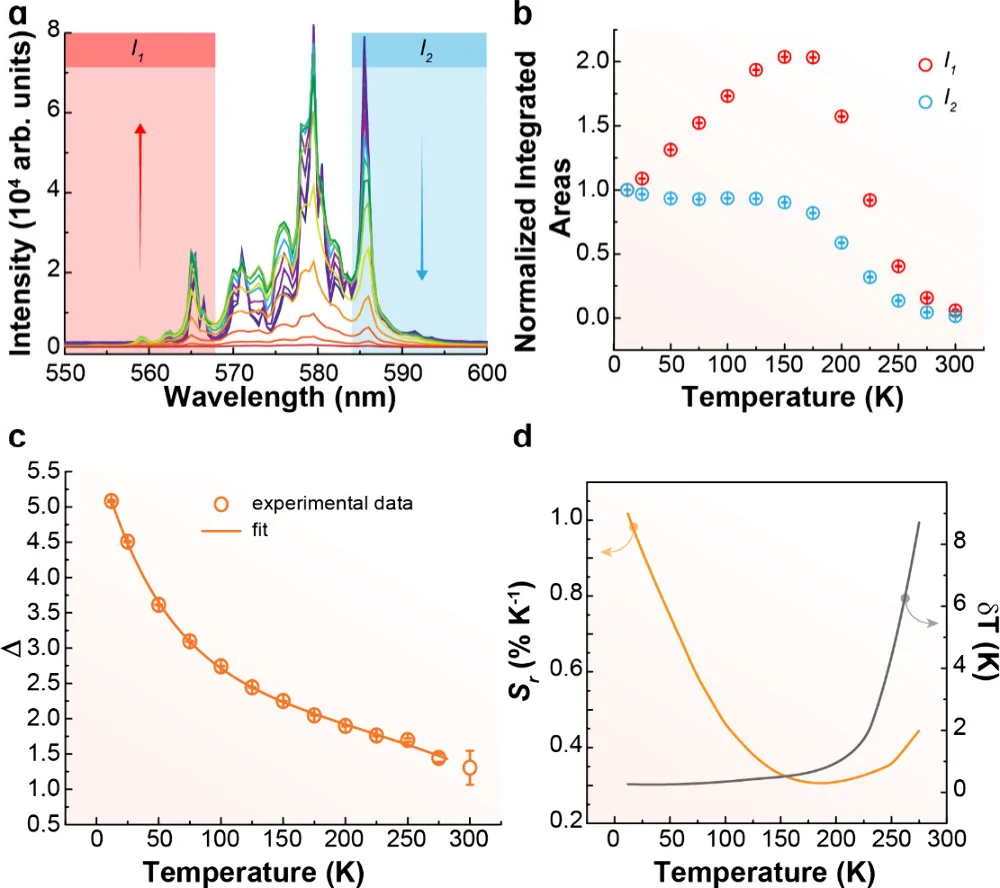
(a) Temperature-dependent emission spectra (12–300 K) of **2**·0.5CH_2_Cl_2_ in the 550–600 nm range, highlighting the spectral regions used to define the thermometric parameter: *I*_1_ (550–567 nm) and *I*_2_ (584–600 nm). (b) Temperature dependence of the normalized integrated emission areas corresponding to *I*_1_ and *I*_2_. (c) Temperature dependence of the thermometric parameter (Δ = *I*_1_/*I*_2_); the solid line corresponds to the fitting of the experimental data within the operative temperature range of 12–275 K. (d) Temperature-dependent relative thermal sensitivity (*S*_*r*_^Δ^) and corresponding temperature uncertainty (*δT* ^Δ^) derived from Δ.

As previously observed for the ^4^F_9/2_→^6^H_15/2_ transition, the emission band exhibits a pronounced splitting (Figure S9). The number of fitted components exceeds that expected from transitions involving only the lowest emitting Kramers doublet, supporting the presence of hot-band contributions from excited sublevels of the ^4^F_9/2_ state. In addition, possible vibronic contributions may further increase the complexity of the observed emission profile.^45^ Consequently, the emission band consists of multiple contributions associated with different emitting sublevels, whose relative population varies with temperature.^16,45^ This results in a non-uniform evolution of the intensity across the band, allowing the identification of regions with opposite trends (Figure 10a), which constitutes the basis for the definition of the thermometric parameter.

In this context, a ratiometric parameter was defined as Δ = *I*_1_/*I*_2_, where *I*_1_ and *I*_2_ are the integrated emission intensities over the spectral ranges of 561–580 nm and 583–600 nm, respectively. The evolution of the normalized integrated areas shows opposite temperature trends, with *I*_1_ increasing at low temperatures and progressively decreasing at higher temperatures, while *I*_2_ exhibits a continuous decrease over the entire temperature range (Figure 10b). As a result, the Δ parameter displays an exponential decrease with temperature. The experimental data were satisfactorily described by a phenomenological exponential function within the operative temperature range of 12–275 K (Figure 10c). The relative thermal sensitivity associated with the thermometric parameter, *S*_*r*_^Δ^, reaches a maximum value of 1% K^–1^ at 12 K. The minimum temperature uncertainty, δ*T* ^Δ^, is 0.2 K at 12 K (Figure 10d). These values lie at the upper end of the range reported for thermometry Dy^3+^ SMMs in the cryogenic regime,^20^ clearly surpassing those observed in Dy^3+^ molecular magnets that also display magnetic thermometric behavior (Table S6).^16,17^

Attempts were also made to combine independent magnetic and luminescent thermometric responses in order to evaluate whether a multiple linear regression (MLR) approach could improve the thermometric performance. Accordingly, the degree of linear correlation between the different thermometric observables was first analyzed using the Pearson correlation coefficient (*R*, eq 4), to determine whether these parameters provide independent thermal information or merely represent correlated manifestations of the same physical process. This analysis is particularly relevant in multiparametric thermometry, since the presence of strong correlations between variables may lead to significant multicollinearity effects, limiting the physical significance of multivariate regression models and reducing the effective dimensionality of the thermometric response space.

The logarithm of the relaxation time, ln­(τ), was considered instead of τ because, within the Orbach regime, τ follows an exponential temperature dependence, and its logarithmic transformation provides the appropriate variable for linear correlation analysis. Conversely, the luminescent thermometric parameter Δ was retained in its original form, since the objective of the present analysis was to evaluate the statistical dependence between the experimentally employed thermometric observables rather than to optimize a regression model through variable transformations.

The Pearson correlation coefficient measures the magnitude and sign of the linear association between two variables and is defined as

$$R_{xy}=\frac{\overset{n}{\underset{i=1}{\sum }}\left(x_{i}-\mathrm{x̅}\right)\left(y_{i}-\mathrm{y̅}\right)}{\sqrt{\overset{n}{\underset{i=1}{\sum }}{\left(x_{i}-\mathrm{x̅}\right)}^{2}}\sqrt{\overset{n}{\underset{i=1}{\sum }}{\left(y_{i}-\mathrm{y̅}\right)}^{2}}}$$ 4

where *x*_*i*_ and *y*_*i*_ correspond to the experimental values of the two variables under analysis, while *x̅* and *y̅* are their respective mean values. The coefficient ranges from −1 to +1, where *r* = +1 corresponds to a perfect positive linear correlation, *r* = −1 to a perfect negative linear correlation, and *r* = 0 indicates the absence of a linear relationship. The Pearson correlation matrix obtained for the thermometric observables 1/χ, Δ, and ln­(τ), calculated over the common temperature interval (12–22 K) in which all three observables are simultaneously available, is shown in Table 2.

**Table 2:** Coefficients of the Correlation Matrix Obtained for the Thermometric Observables 1/χ, Δ, and ln­(τ)

	**1**/**χ**	**Δ**	**ln**(***τ***)
**1**/**χ**	1.000000	0.998052	–0.998195
**Δ**	0.998052	1.000000	–0.999996
**ln**(**τ**)	–0.998195	–0.999996	1.000000

The obtained coefficients reveal an almost perfect linear correlation between all observables (absolute value of the Pearson correlation coefficient is near 1), demonstrating that the thermometric parameters are strongly collinear and predominantly governed by the same thermally activated physical process. In particular, the negative correlations involving ln­(τ) indicate that this parameter evolves oppositely with temperature relative to 1/χ and Δ, while the positive correlation between 1/χ and Δ reflects their similar thermal evolution.

Under these conditions, the application of a conventional MLR model becomes statistically ambiguous, since the regression coefficients turn highly sensitive to small experimental fluctuations and lose a clear individual physical meaning due to multicollinearity effects.

It is important to emphasize that this limitation does not arise from the thermometric observables themselves, but from their statistical redundancy. Since the three observables are governed by the same thermally activated mechanism, they do not provide independent thermal information. Consequently, combining them within a linear multivariate framework does not necessarily improve the thermometric performance over the best individual thermometric parameter. In contrast, multivariate thermometry is expected to be advantageous only when the different observables probe complementary and partially independent physical processes, thereby contributing non-redundant information to the temperature prediction.

To further evaluate the effective dimensionality of the thermometric response, a principal component analysis (PCA) was performed (see details in the Supporting Information). The analysis revealed that the first principal component accounts for virtually all the variance of the dataset (>99%), confirming that the thermometric response space is essentially one-dimensional. Therefore, PCA does not provide additional physical insight beyond confirming that the different thermometric observables correspond to correlated manifestations of the same dominant thermal mechanism. The excellent predictive performance obtained from PC1 mainly reflects statistical averaging and noise reduction rather than the incorporation of additional independent thermometric information.

From a practical perspective, luminescence thermometry remains the most accessible read-out strategy owing to its non-contact nature, high spatial resolution, and relatively simple instrumentation compared with magnetic thermometry based on *ac* susceptibility measurements. In this context, magnetic thermometry should be regarded as a complementary functionality that enriches the characterization of multifunctional materials rather than as a direct alternative to optical thermometry. The present correlation analysis further suggests that future multiparametric thermometers should preferentially combine sensing channels governed by independent physical mechanisms, thereby maximizing the amount of complementary thermal information while avoiding statistical redundancy.

Nevertheless, despite these rather limited outcomes, it is worth noting that the degree of linear correlation has been evaluated for the first time in a dual luminescent–magneto thermometer.

## Conclusions

[Dy­(L^N6prop^)­(CH_3_COO)­(OSiPh_3_)]­(BPh_4_)·0.5CH_2_Cl_2_ (**2**·0.5CH_2_Cl_2_) behaves as a single-ion magnet with an energy barrier of 465 K, consistent with *ab initio* calculations and luminescence measurements. Comparison of its magnetic properties with those of structurally related Dy^3+^ SIMs, based on *N*_6_ macrocycles incorporating triphenylsilanolate and acetate as auxiliary ligands, highlights that the strength of the equatorial ligand field seems to play a critical role in the anisotropic energy barrier.

In addition, **2**·0.5CH_2_Cl_2_ acts as a dual-function thermometer, integrating luminescent response with temperature-dependent magnetic behavior. It delivers luminescent thermometric performance among the best reported for non-field-induced SMMs, alongside highly efficient magnetic thermometry in the cryogenic regime, predominantly governed by the relaxation time τ. Thus, **2**·0.5CH_2_Cl_2_ is a bifunctional molecular material, integrating single-molecule magnetism with efficient dual-mode luminescent and magnetic thermometry.

The feasibility of integrating optical and magnetic observables through a conventional multiple linear regression (MLR) approach was also examined. However, evaluation of the interdependence between the thermometric parameters renders pronounced multicollinearity, precluding the applicability of a conventional MLR approach. To further probe the intrinsic dimensionality of the thermometric response space, a principal component analysis (PCA) was performed. The results show that both optical and magnetic signals arise from a strongly correlated thermal response rather than independent information channels. Consequently, PCA confirms that no improvement in predictive performance is gained by combining the observables within a linear multivariate framework.

Despite these limitations, it is noteworthy that this work provides the first quantitative assessment of the linear correlation between luminescent and magnetic thermometric observables in a dual luminescent–magneto thermometer, establishing a benchmark for future studies aimed at true multimodal thermal readout strategies.

## Supplementary Material



## Notes

Experimental details of PCA calculations, additional figures, tables and SHAPE calculations (PDF)
