# Supplementary material for: A Dy3+ Single-Molecule Magnet as a Dual-Mode Luminescent and Magnetic Thermometer
Source: Inorg Chem. 2026 Sep 11;65(37):21572–82. doi: 10.1021/acs.inorgchem.6c02533 (PMC13599389; doi:10.1021/acs.inorgchem.6c02533)
Supplement: Supplementary file 1 [file ic6c02533_si_001.pdf]

Supplementary material

A Dy^{3+} Single-Molecule Magnet as a Dual-Mode Luminescent and Magnetic Thermometer

Paula Oreiro-Martínez,[‡,ε] Cristina González-Barreira,[‡] Julio Corredoira-Vázquez[‡,ε,] Ana M. García-Deibe,[‡] Jesús Sanmartín-Matalobos,[‡,ε] Carlos D. S. Brites,[§] Luis D. Carlos,[§] Daniel Aravena[Ψ] and Matilde Fondo[‡,ε,]**

[‡] Departamento de Química Inorgánica, Facultade de Química, Universidade de Santiago de Compostela, 15782 Santiago de Compostela, Spain. E-mail: matilde.fondo@usc.es; julio.corredoira.vazquez@usc.es

[ε] Institute of Materials (iMATUS), Universidade de Santiago de Compostela, 15782 Santiago de Compostela, Spain

[§] Phantom-g, CICECO–Aveiro Institute of Materials, Physics Department University of Aveiro, 3810-193, Aveiro, Portugal

[Ψ] Departamento de Química de los Materiales, Facultad de Química y Biología, Universidad de Santiago de Chile, Casilla 40, Correo 33 Santiago, Chile

Corresponding Authors

Matilde Fondo, E-mail: matilde.fondo@usc.es

Julio Corredoira-Vázquez, E-mail: julio.corredoira.vazquez@usc.es

1. X-RAY CHARACTERIZATION

Table S1. Crystal data and structure refinement for **2**·$0.5CH_2Cl_2$. page S3

Table S2. Main bond distances (Å) and angles (°) for **2**·$0.5CH_2Cl_2$. page S4

Table S3. SHAPE v2.1. Continuous Shape Measures calculation (c) 2013. Electronic Structure Group, Universitat de Barcelona. page S4

Figure S1. Comparative powder X-ray diffractograms for **2**·$0.5CH_2Cl_2$. Blue: calculated diffractogram using the data obtained from single X-ray diffraction studies; green: experimental diffractogram. page S5

2. MAGNETIC STUDIES

Figure S2. $\chi_M T$ *vs* T graph for **2**·$0.5CH_2Cl_2$. Inset: $M/N\mu_B$ *vs* H at 2 K. page S6

Figure S3. Temperature dependence of χ''_M for **2**·$0.5CH_2Cl_2$ (H_{dc} = 0) at different frequencies. page S6

Figure S4. Cole-Cole graph for **2**·$0.5CH_2Cl_2$ at H_{dc} = 0 in the range 2-22 K. page S7

Table S4. Some selected structural and magnetic parameters for Dy^{3+} complexes with N_6 macrocycles and acetate and triphenylsilanolate as auxiliary ligands. page S8

Figure S5. Field dependence of the magnetic relaxation time at 5 K for **2**·$0.5CH_2Cl_2$. page S9

Figure S6. Temperature dependence of χ''_M for **2**·$0.5CH_2Cl_2$ at different frequencies in H_{dc} = 1000 Oe. page S9

Figure S7. Cole-Cole graph for **2**·$0.5CH_2Cl_2$ at H_{dc} = 1000 Oe in the range 7-28 K. page S9

3. LUMINISCENCE AND THERMOMETRIC STUDIES

Figure S8. Excitation spectra of **2**·$0.5CH_2Cl_2$ in the 12-300 K temperature range, monitoring the $^4F_{9/2} \rightarrow {}^6H_{13/2}$ transition. page S10

Figure S9. Deconvolution of the $^4F_{9/2} \rightarrow {}^6H_{15/2}$ transition of **2**·$0.5CH_2Cl_2$ (11 K), excited at 348 nm. The green circles depict the experimental data, and the red line represents the envelope fit using multi-Gaussian functions. page S10

Table S5. Energy of the Kramers doublets (KDs) obtained from spectroscopic measurements of complex **2**·$0.5CH_2Cl_2$. Page S11

Table S6. Some magneto and thermometric parameters for combined magneto-luminescent thermometers with SMM behaviour. Page S11

Principal component analysis (PCA) Page S12

Table S7. The first principal component (PC1) and predicted *vs* real temperatures with thermal sensitivities. Page S14

Figure S10. Temperature dependence of $ln(\tau)$. The solid line represents the linear fit in the 7–22 K range ($r^2 > 0.997$). page S14

References page S15

1. X-RAY CHARACTERIZATION

Table S1. Crystal data and structure refinement for **2**·$0.5CH_2Cl_2$.

Empirical formula	$C_{64.5}H_{61}BClDyN_6O_3Si$
Formula weight	1205.04
Crystal system	Triclinic
Space group	*P*-1
Wavelength	0.71073
Crystal size (mm^3)	0.12 x 0.08 x 0.02
Shape, color	Plate, colorless
T (K)	100
a (Å)	13.7862(17)
b (Å)	14.3756(19)
c (Å)	16.242(2)
α (º)	80.675(4)
β (º)	74.942(4)
γ (º)	68.973(4)
Volume (Å^3)	2893.0(7)
Z	2
Absorption coefficient (mm^{-1})	1410
Reflections collected	15724
Theta range for data collection	2.496 to 26.508º
Completeness to theta = 25.242	76.4 %
Independent reflections	8956 {[R_{int} = 0.0654]
Data / restraints / parameters	8956 / 12 / 713
Final R indices [I>2sigma(I)]	R_1 = 0.0854; wR_2 =0.1768
R indices (all data)	R_1 = 0.1194; wR_2 =0.1948

Table S2. Main bond distances (Å) and angles (°) for **2**·0.5CH_2Cl_2.

Dy1-O1	2.124(8)	Dy1-N3	2.566(9)
Dy1-O3	2.395(7)	Dy1-N4	2.652(9)
Dy1-O2	2.428(7)	Dy1-N5	2.589(10)
Dy1-N1	2.660(8)	Dy1-N6	2.589(9)
Dy1-N2	2.595(9)		
O1-Dy1-O3	150.7(3)	N5-Dy1-N2	168.5(3)
O1-Dy1-O2	154.8(3)	N2-Dy1-N1	61.2(3)
N3-Dy1-N6	149.9(3)	O3-Dy1-O2	54.5(2)

Table S3. SHAPE v2.1. Continuous Shape Measures calculation (c) 2013. Electronic Structure Group, Universitat de Barcelona

Coordination number 9 geometries

MFF-9	13	C_s	Muffin
HH-9	12	C_{2v}	Hula-hoop
JTDIC-9	11	C_{3v}	Tridiminished icosahedron J63
TCTPR-9	10	D_{3h}	Spherical tricapped trigonal prism
JTCTPR-9	9	D_{3h}	Tricapped trigonal prism J51
CSAPR-9	8	C_{4v}	Spherical capped square antiprism
JCSAPR-9	7	C_{4v}	Capped square antiprism J10
CCU-9	6	C_{4v}	Spherical-relaxed capped cube
JCCU-9	5	C_{4v}	Capped cube J8
JTC-9	4	C_{3v}	Johnson triangular cupola J3
HBPY-9	3	D_{7h}	Heptagonal bipyramid
OPY-9	2	C_{8v}	Octagonal pyramid
EP-9	1	D_{9h}	Enneagon

2·0.5CH_2Cl_2

Structure [ML_9]	**MFF-9**	HH-9	JTDIC-9	TCTPR-9	JTCTPR-9	**CSAPR-9**
	**2.012**,	8.895,	10.756,	2.863,	4.393,	**1.909**,

JCSAPR-9	CCU-9	JCCU-9	JTC-9	HBPY-9	OPY-9	EP-9
2.680,	5.410,	6.596,	15.941,	15.998,	24.017,	36.365

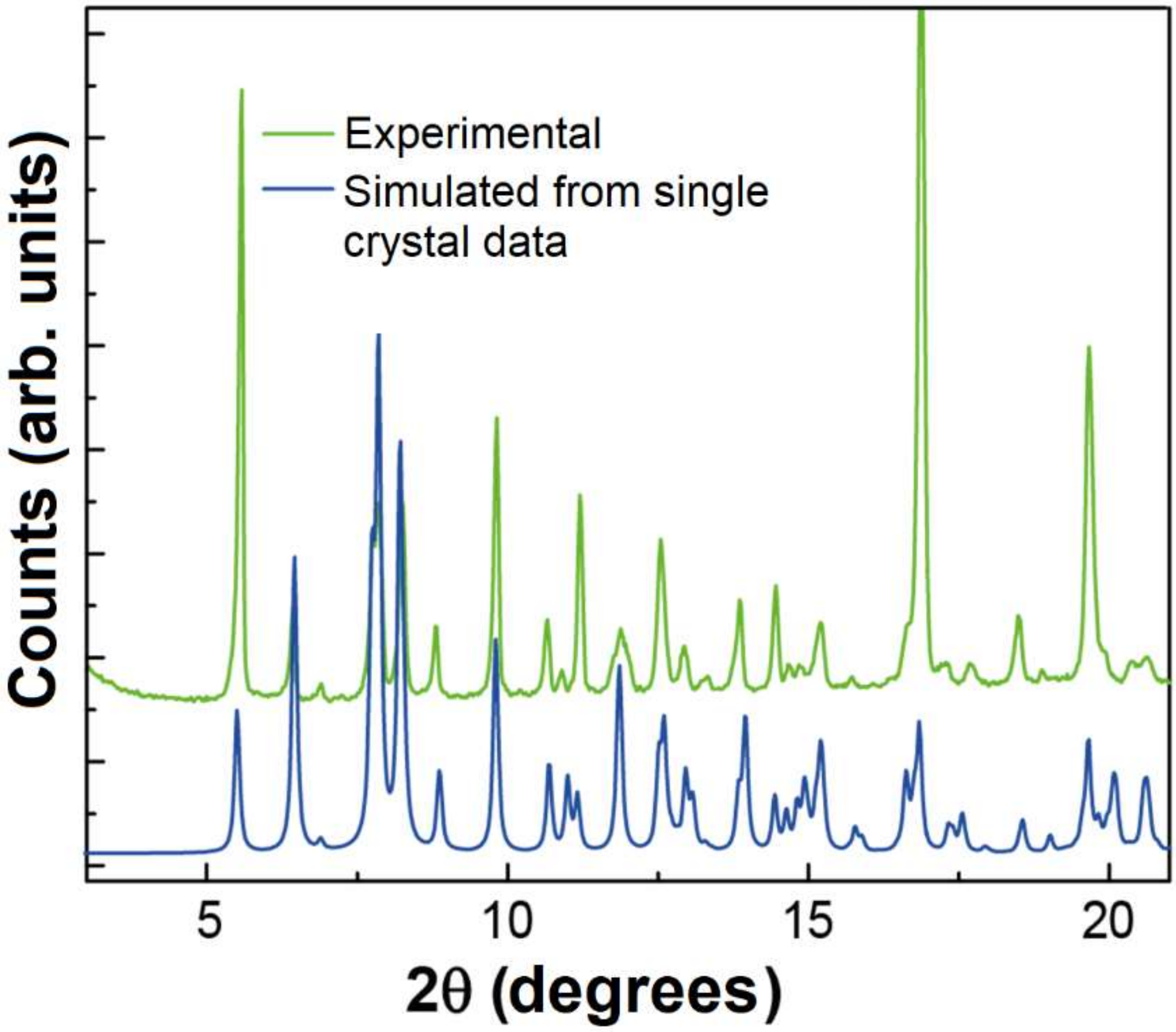

Figure S1. Comparative powder X-ray diffractograms for **2**·$0.5CH_2Cl_2$. Blue: calculated diffractogram using the data obtained from single X-ray diffraction studies; green: experimental diffractogram.

2. MAGNETIC STUDIES

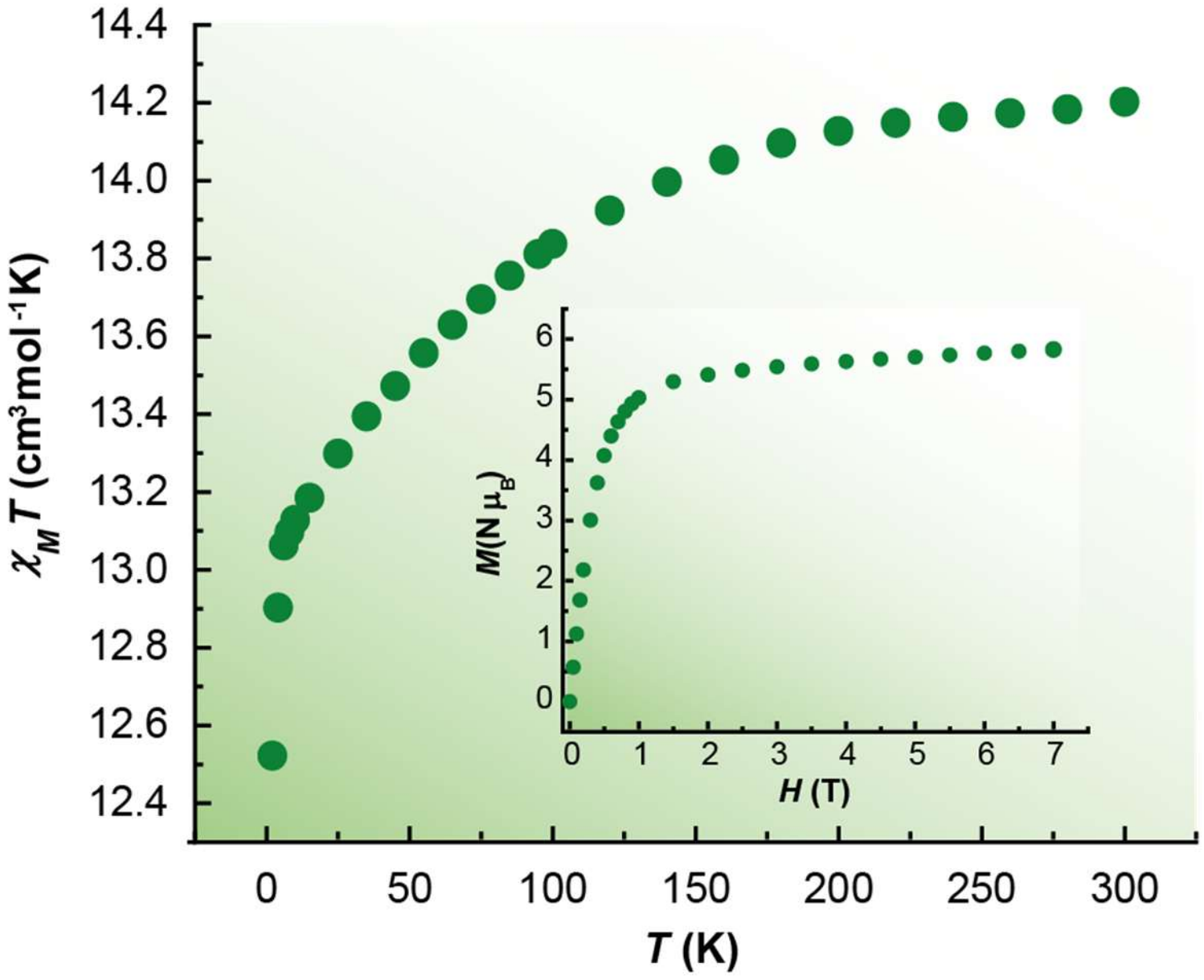

Figure S2. $\chi_M T$ *vs* T graph for **2**·0.5CH_2Cl_2. Inset: $M/N\mu_B$ *vs* H at 2 K.

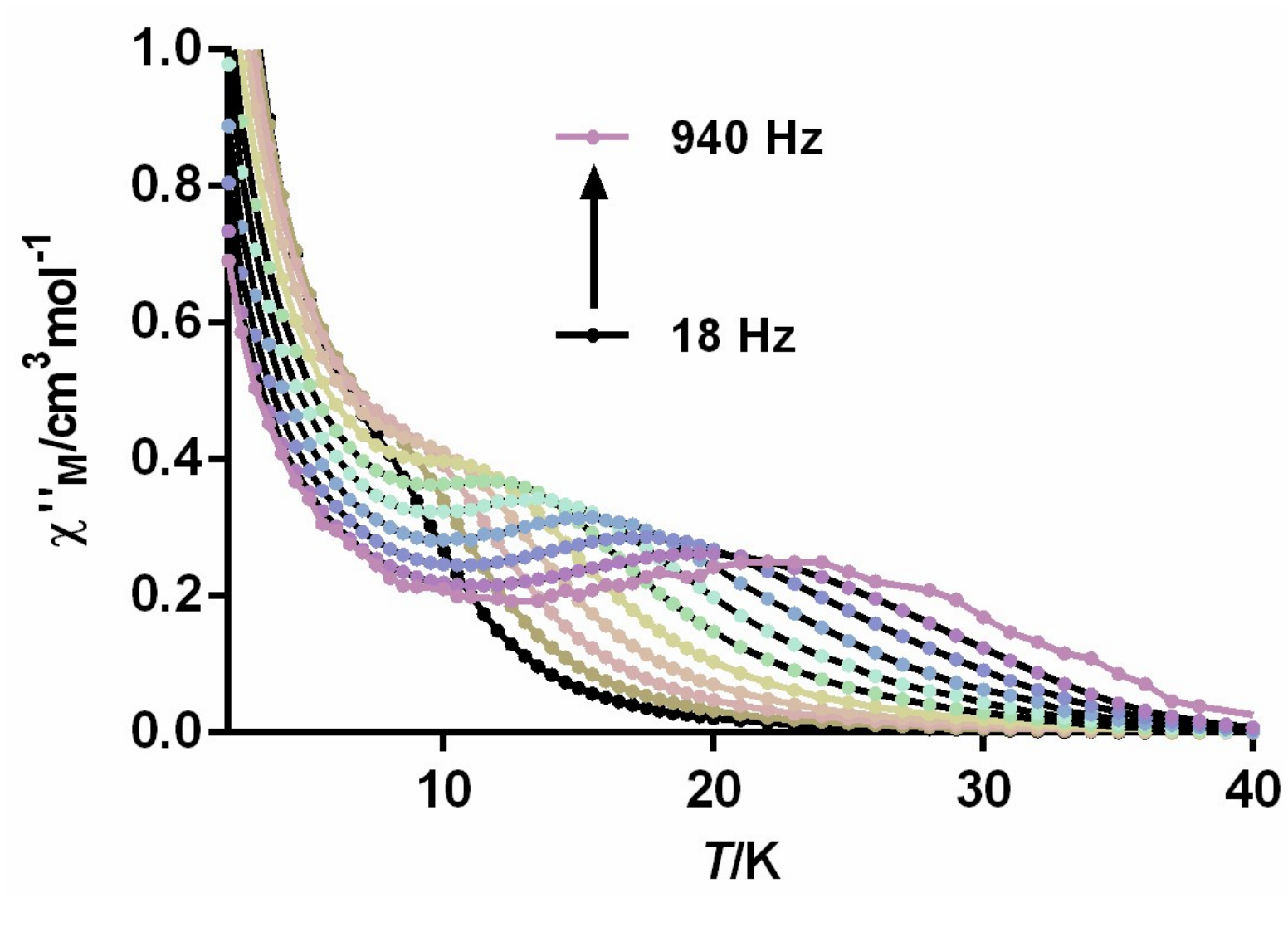

Figure S3. Temperature dependence of χ''_M for **2**·0.5CH_2Cl_2 (H_{dc} = 0) at different frequencies.

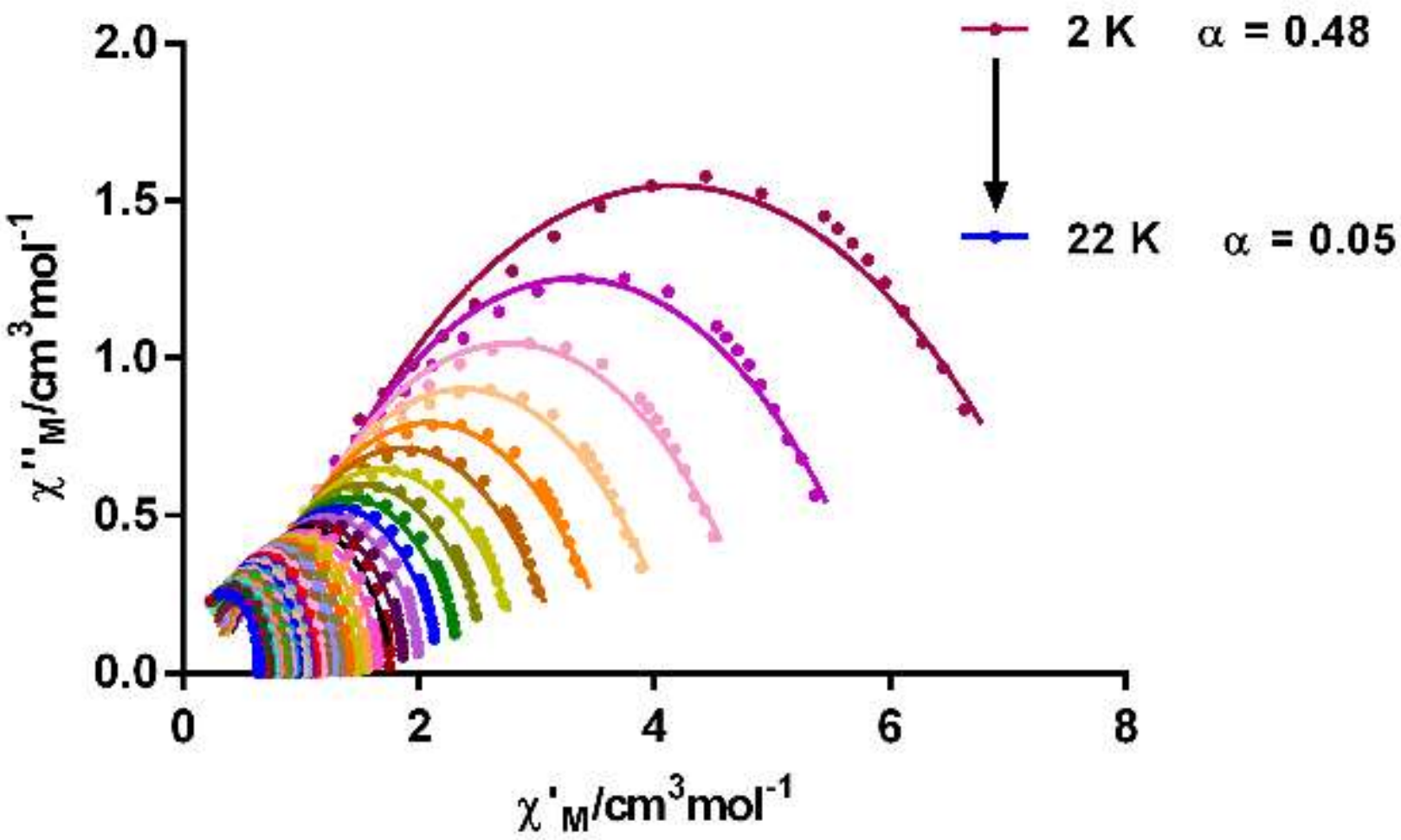

Figure S4. Cole-Cole graph for **2**·$0.5CH_2Cl_2$ at H_{dc} = 0 in the range 2-22 K.

Table S4. Some selected structural and magnetic parameters for Dy^{3+} complexes with N_6 macrocycles and acetate and triphenylsilanolate as auxiliary ligands.

**Metal complex**[a]	**Geometry**[b]	**Dy-N (Å)**	**Dy- O_{Ph3SiO} (Å)**	**Dy-OAc (Å)**	**Dev. N-plane**[c]	**Dev. Dy-plane**[d]	U_{eff} **(K)/** H_{dc} [e]	**Mechanism**[f]	**Ref.**
$[Dy(L^{N6enMe})(CH_3COO)(Ph_3SiO)](PF_6)$	HH	2.558(8)-2.603(6)	2.102(7)	2.402(7)	0.230-0.463	0.191	36.6/0.5	O + R + QTM	[1]
$[Dy(L^{N6phenMe})(CH_3COO)(Ph_3SiO)](BPh_4)$	HH	2.571(2)-2.622(3)	2.110(2)	2.437(2)-2.456(2)	0.255-0.559	0.075	150.1/0	O + R + QTM	[2]
$[Dy(L^{N6prop})(CH_3COO)(Ph_3SiO)](BPh_4)$	CSAPR	2.595(9)-2.660(8)	2.124(8)	2.395(7)-2.428(7)	0.140-0.766	0.110	150.0/0	O + R + QTM	This work

[a] ligands depicted below and solvent molecules omitted for clarity; [b] HH: Hula-hoop, CSAPR: spherical capped square antiprism; [c] distance from the equatorial *N*-atoms to the mean calculated N_6 plane in Å; [d] distance from the Dy^{3+} ions to the mean calculated N_6 plane in Å; [e] applied H_{dc} in KOe; [f] relaxation mechanism: O = Orbach, R: Raman, QTM = Quantum Tunnel

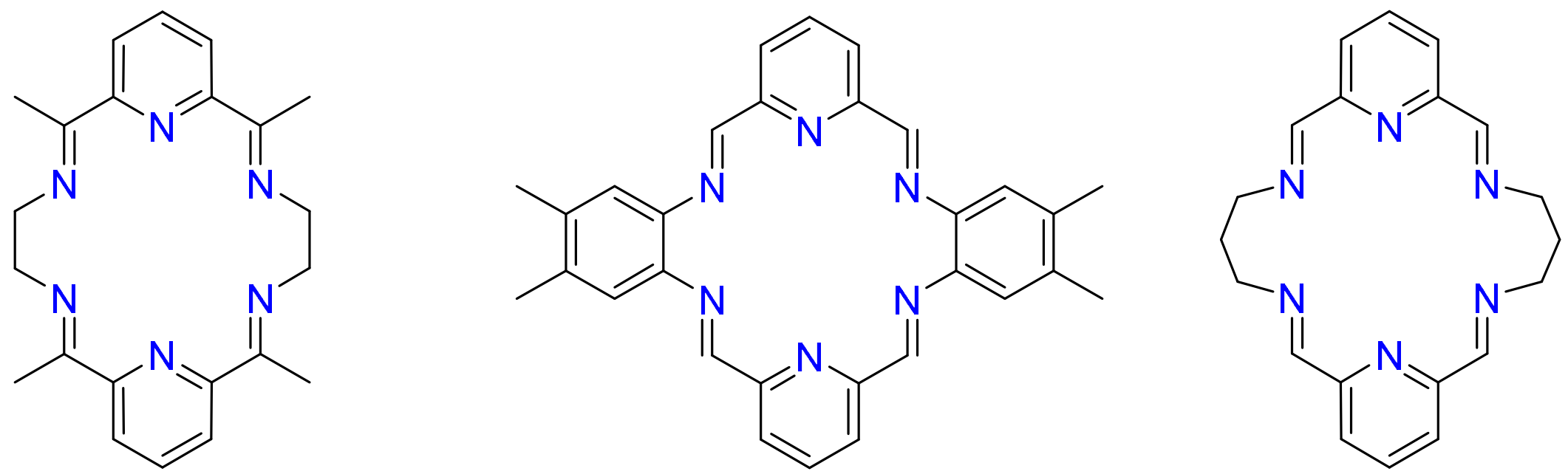

$\mathbf{L^{N6enMe}}$ $\mathbf{L^{N6phenMe}}$ $\mathbf{L^{N6prop}}$

Ligands in Table S4

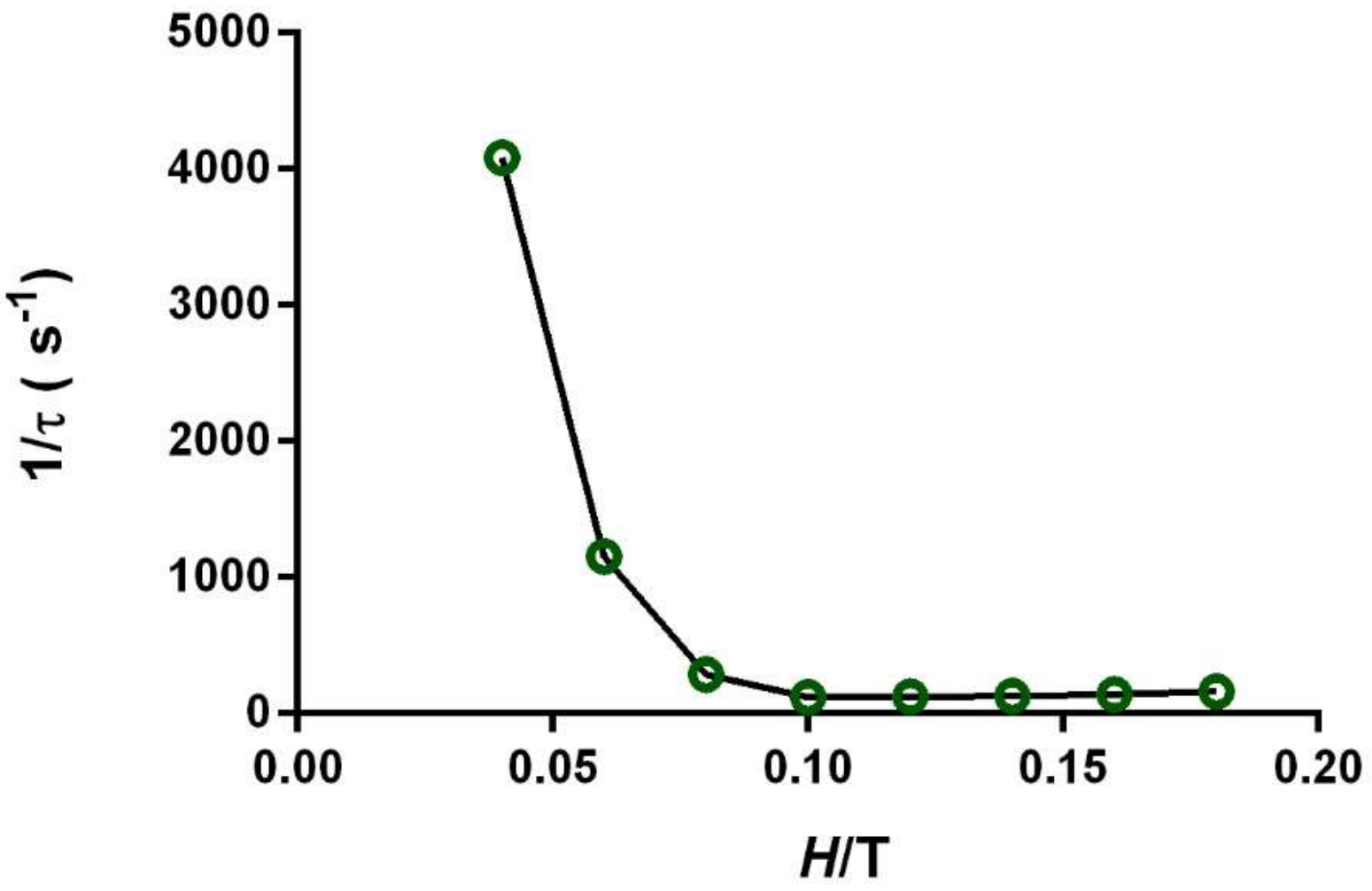

Figure S5. Field dependence of the magnetic relaxation time at 5 K for **2**·0.5CH_2Cl_2.

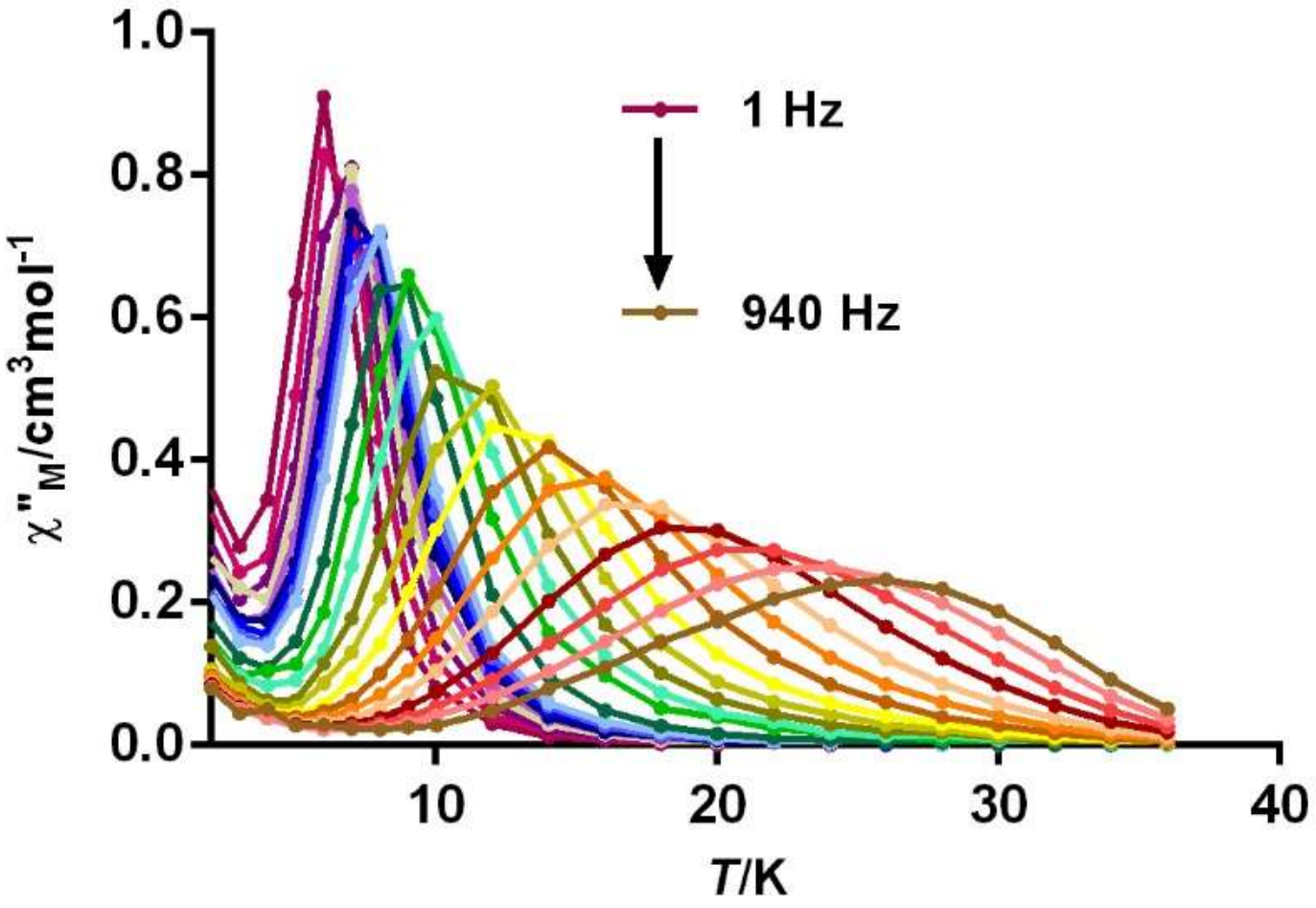

Figure S6. Temperature dependence of χ''_M for **2**·0.5CH_2Cl_2 at different frequencies in H_{dc} = 1000 Oe.

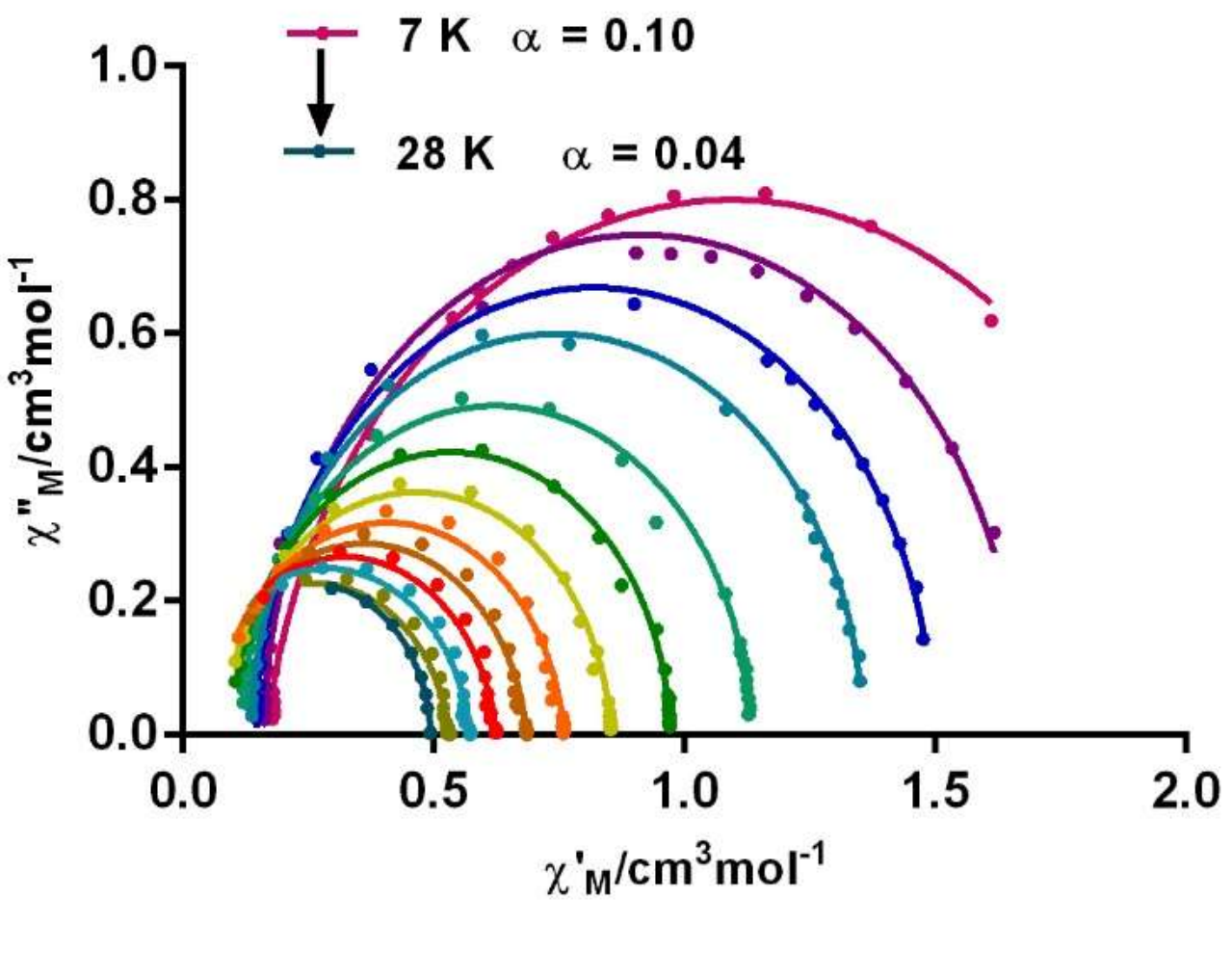

Figure S7. Cole-Cole graph for **2**·0.5CH_2Cl_2 at H_{dc} = 1000 Oe in the range 7-28 K.

3. LUMINISCENCE AND THERMOMETRIC STUDIES

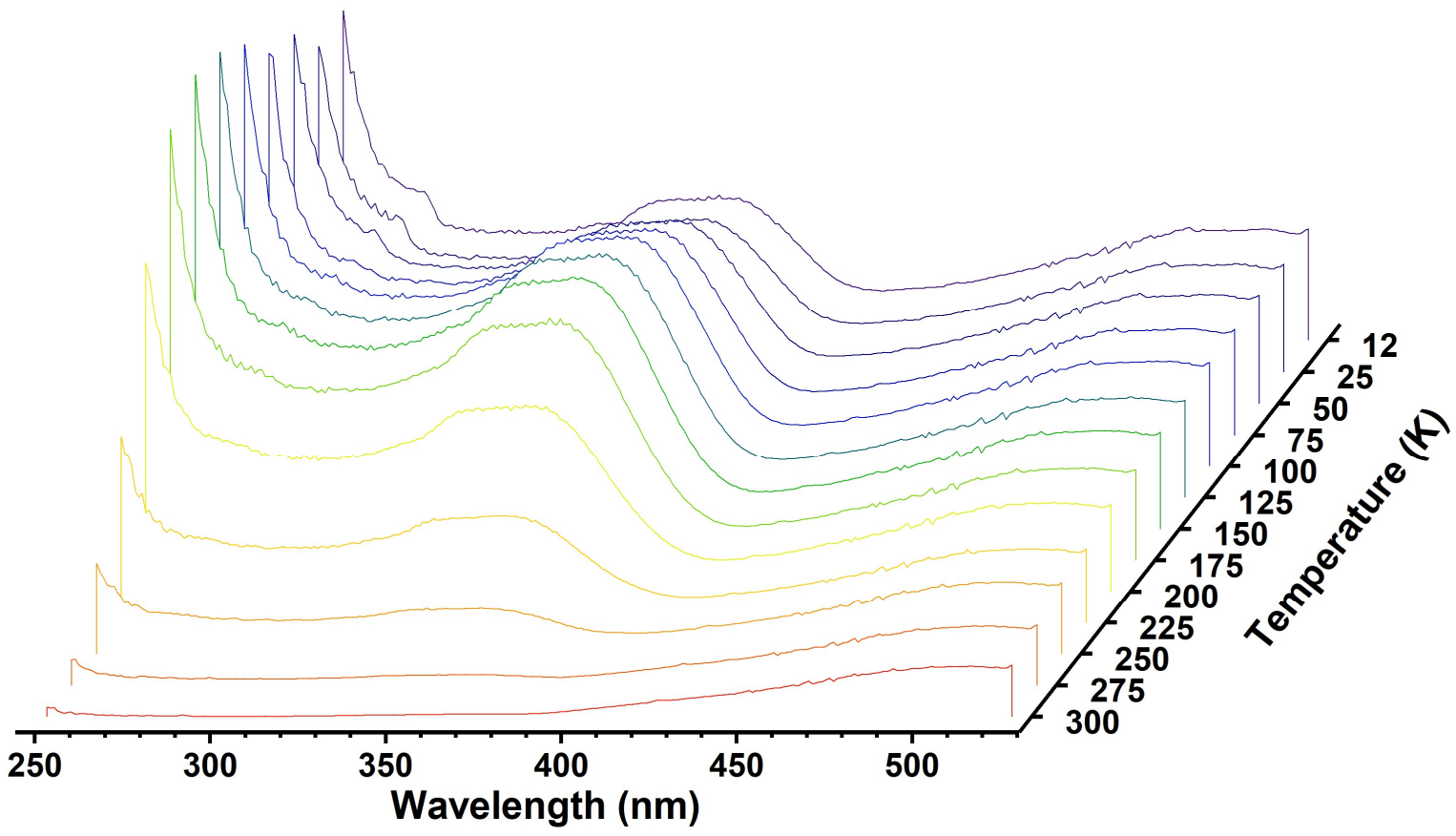

Figure S8. Excitation spectra of **2**·0.5CH_2Cl_2 in the 12-300 K temperature range, monitoring the $^4F_{9/2} \rightarrow {}^6H_{13/2}$ transition.

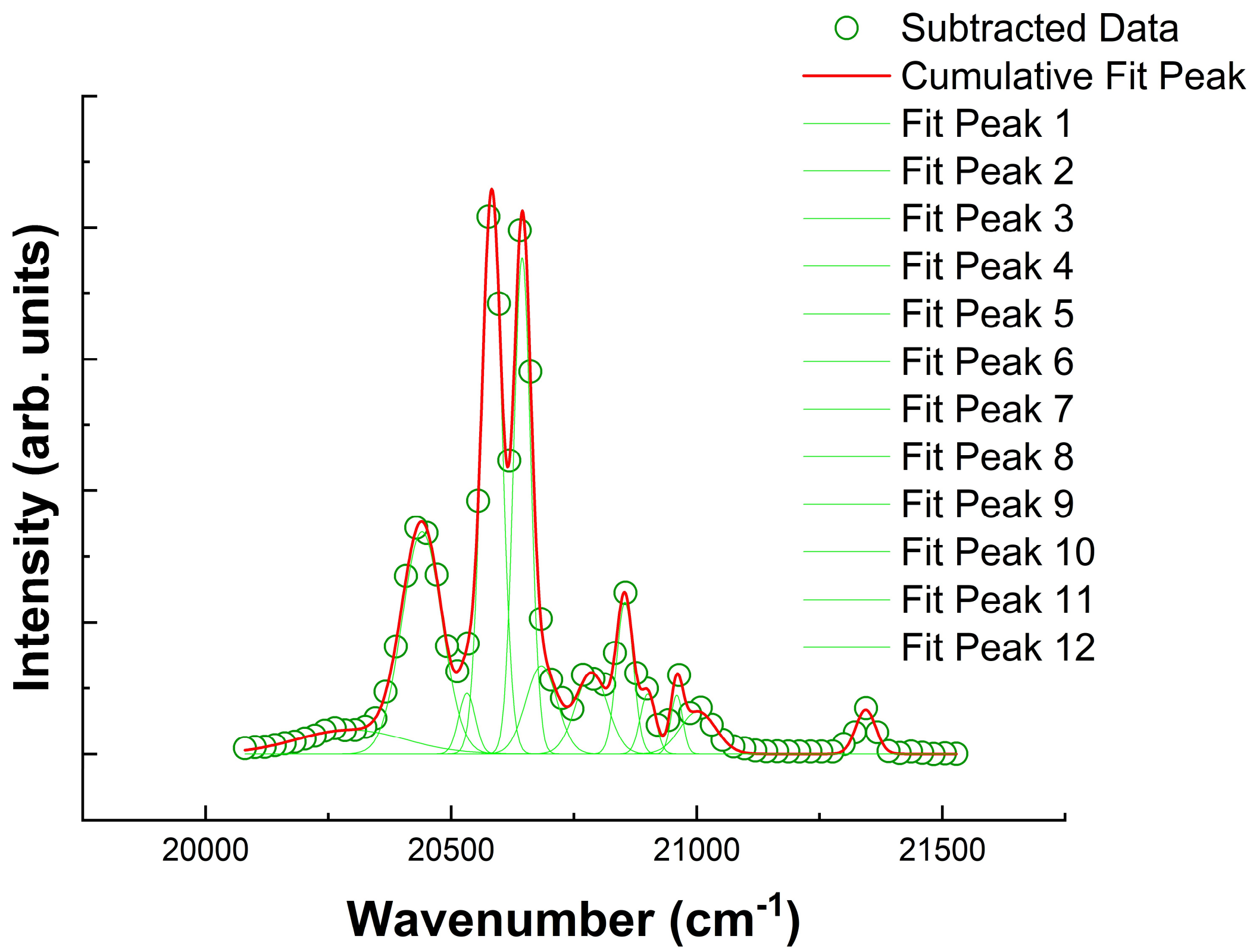

Figure S9. Deconvolution of the $^4F_{9/2} \rightarrow {}^6H_{15/2}$ transition of **2**·0.5CH_2Cl_2 (11 K), excited at 348 nm. The green circles depict the experimental data, and the red line represents the envelope fit using multi-Gaussian functions.

Table S5. Energy of the Kramers doublets (KDs) obtained from spectroscopic measurements of complex **2**·0.5CH_2Cl_2.

Peak	Centered (cm^{-1})	$\|E_0 - E_n\|$ (cm^{-1})	Ab initio	Assignment
1	21343.9	0	0.0	KD1
2	21012.3	331.7	324.6	KD2
3	20959.3	384.7	-	HOT
4	20900.2	443.8	-	HOT
5	20853.8	490.2	496.5	KD3
6	20766.1	577.9	591.4	KD4
7	20683.8	660.2		HOT
8	20644.5	699.4	719.4	KD5
9	20582.7	761.3	756.0	KD6
10	20542.2	801.8	783.8	KD7
11	20440.4	903.6	903.2	KD8
12	20295.5	1048.42805	-	HOT

Table S6. Some magneto and thermometric parameters for combined magneto-luminescent thermometers with SMM behaviour.

		Luminescence themometry[a]		Magnetometry: 1/χ[a]		Magnetometry: τ[a]		
Compound*	U_{eff} (K)	S_m/%K^{-1}	T_{uncert}/K	$S_m^{1/\chi}$/%K^{-1} (T/K)	T_{uncert}/K	S_m^{τ}/%K^{-1} (T/K)	T_{uncert}/K	Ref.
[Dy(bbpen)Cl]	813.3	0.3 (225)	0.4 (nr)	0.06 (15)	nr	*ca*. 0.3 (50)	nr	3
[Dy_2(bbpen_Me)$_2$(CA)]	585.6	0.25 (68)	0.03 (nr)	9.90 (10)	<0.11 (nr)	11.9 (16)	<0.011 (nr)	4
**2**·0.5CH_2Cl_2	150.0	1 (12)	0.2 (12)	9.3 (10)	0.3 (10)	20 (9)	0.03	This work

*Solvate molecules are omitted for clarity; H_2bbpen =N,N'-bis(2-hydroxybenzyl)-N,N'-bis(2-methylpyridyl)ethyl-enediamine; H_2bbpen = N,N′-bis(2-hydroxy-5-methylbenzyl)-N,N′-bis(2-methylpyridyl)ethylenediamine; CA = chloranilic acid. [a] S_m means maximum sensitivity, in parentheses

the temperature at which it is achieved, in K; T_{uncert} means minimum temperature uncertainty, in parentheses the temperature at which it is achieved. nr = not reported

Principal component analysis (PCA)

The performance of the PCA-based thermometric model was evaluated by correlating the first principal component (PC1) with temperature through linear regression.

To evaluate the effective dimensionality of the thermometric dataset, a principal component analysis (PCA) was performed using the thermometric observables $1/\chi$, Δ, and $\ln(\tau)$. Prior to the analysis, all variables were standardized according to:

$$Z_i = \frac{X_i - \bar{X}_i}{\sigma_i}$$

where X_i corresponds to the experimental value of a given thermometric parameter, $\bar{X}_i$ is its mean value, and σ_i its standard deviation. This normalization ensures that all variables contribute equally to the analysis, independently of their absolute scales.

PCA transforms the original correlated variables into a new set of orthogonal variables, called principal components, which are linear combinations of the standardized parameters. The first principal component (PC1) is defined as the direction in multidimensional space that maximizes the variance of the dataset and can be expressed as:

$$PC1 = a_1 Z_{1/\chi} + a_2 Z_{\Delta} + a_3 Z_{\ln(\tau)}$$

where a_1, a_2, and a_3 are the loading coefficients obtained from the eigenvectors of the covariance matrix.

For the present dataset, the loading coefficients associated with PC1 were found to be approximately:

$$PC1 \approx -0.577\, Z_{1/\chi} + 0.577\, Z_{\Delta} + 0.577\, Z_{\ln(\tau)}$$

indicating that the three observables contribute almost equally to the dominant thermal component, with $1/\chi$ volving oppositely relative to Δ and $\ln(\tau)$. The equal magnitude of the coefficients reflects the strong correlation between the thermometric observables and confirms that the system is effectively governed by a single thermally activated mechanism.

The temperature calibration was subsequently obtained by fitting the experimental temperature values as a linear function of PC1:

$$T = \alpha\, PC1 + \beta$$

where α and β are the regression parameters obtained from the linear fit. The resulting model provides an accurate reconstruction of the experimental temperatures with residual errors below ± 0.26K throughout the analyzed temperature range.

The predicted temperatures (T_{pred}) obtained from the calibration model were compared with the experimental values (T_{real}), and the corresponding residuals ($T_{\mathrm{real}} - T_{\mathrm{pred}}$) were analyzed (Table S6).

The PCA-based calibration exhibits excellent predictive performance over the entire temperature range, with residual errors remaining below ± 0.26K. The residual distribution does not reveal systematic deviations, confirming the robustness and linearity of the PC1-temperature relationship. The relative thermal sensitivity associated with the PCA-derived thermometric parameter decreases smoothly from 3.7 % K^{-1} at 12 K to 2.7 % K^{-1} at 22 K, remaining consistently higher than the values obtained for the individual luminescent parameter Δ.

Despite the excellent predictive capability of the PCA model, the analysis confirms that the thermometric dataset is effectively governed by a single latent thermal variable. Therefore, the improvement obtained through PCA mainly reflects statistical averaging and noise reduction effects rather than the emergence of additional independent thermometric information.

Table S7. The first principal component (PC1) and predicted *vs* real temperatures with thermal sensitivities.

Temperature (K)	PC1	T_{pred} (K)	Residuals (K)	Sr (%.K^{-1})
12.00	5.12	11.74	-0.26	3.70
12.50	5.23	12.32	-0.18	3.60
13.00	5.34	12.93	-0.07	3.50
13.50	5.44	13.45	-0.05	3.50
14.00	5.55	14.04	0.04	3.40
14.50	5.66	14.62	0.12	3.30
15.00	5.75	15.10	0.10	3.30
15.50	5.85	15.63	0.13	3.20
16.00	5.96	16.19	0.19	3.20
16.50	6.04	16.64	0.14	3.10
17.00	6.13	17.13	0.13	3.10
17.50	6.24	17.67	0.17	3.00
18.00	6.33	18.19	0.19	3.00
18.50	6.37	18.39	-0.11	3.00
19.00	6.47	18.93	-0.07	2.90
19.50	6.55	19.36	-0.14	2.90
20.00	6.67	19.97	-0.03	2.80
21.00	6.85	20.92	-0.08	2.70
22.00	7.01	21.79	-0.21	2.70

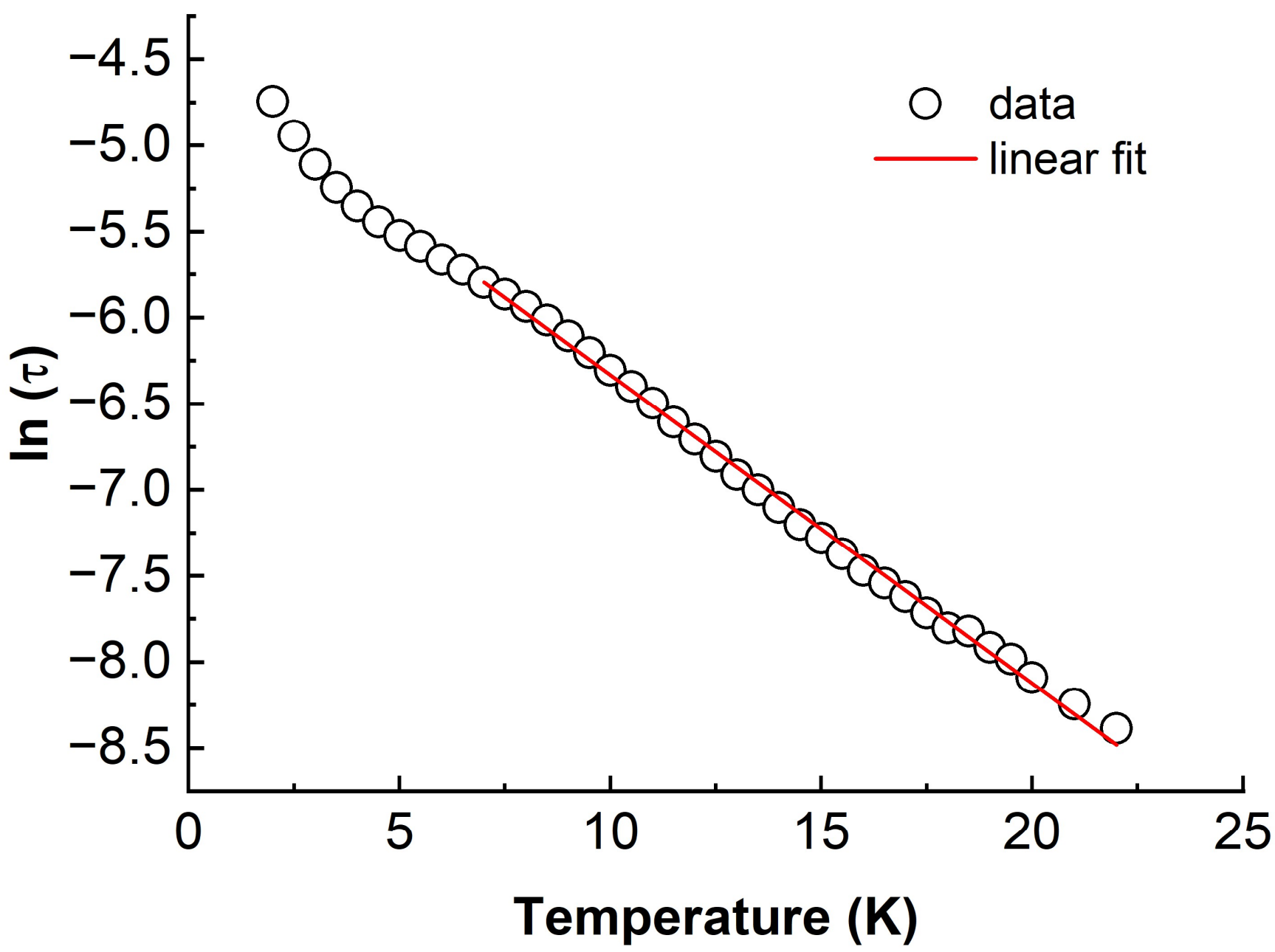

Figure S10. Temperature dependence of $ln(\tau)$. The solid line represents the linear fit in the 7-22 K range ($r^2 > 0.997$).

REFERENCES

(1) Zhong, X.; Rana, R.; Luo, T.-K.; Fang Wu, Y.; Peng, Y.; Liu, S.-J.; Rajaraman, G.; Wen, H.-R. Systematically study of ligands field effect on a pseudo-D_{6h} Dy(III) single-ion magnet with axially asymmetric ligands. *J. Mol. Struct.* **2025**, *1338*, 142270. DOI: 10.1016/j.molstruc.2025.142270.

(2) Zhong, X.; Li, D.-Y.; Cao, C.; Luo, T.-K.; Hu, Z.-B.; Peng, Y.; Liu, S.-J.; Zheng, Y.-Z.; Wen, H.-R. Effect of substituents in equatorial hexaazamacrocyclic schiff base ligands on the construction and magnetism of pseudo D_{6h} single-ion magnets. *Inorg. Chem.* **2024**, *63* (46), 21909-21918. DOI: 10.1021/acs.inorgchem.4c03012.

(3) Zanella, S.; Aragon-Alberti, M.; Brite, C. D. S.; Salles, F.; Carlos, L. D.; Long, J. Luminescent single-molecule magnets as dual magneto-optical molecular thermometers. *Angew. Chem. Int. Ed.* **2023**, *62* (35), e202306970. DOI: https://doi.org/10.1002/anie.202306970.

(4) Shome, S.; Maurya, N. C.; Adarsh, K. V.; Konar, S. Dy-SMM for standalone magneto thermometry and opto-magnetic temperature sensing via multiple linear regression. *Inorg. Chem.* **2025**, *64* (49), 23990-23998. DOI: 10.1021/acs.inorgchem.5c03949.
